# Diminished socially selective neural processing in 5‐month‐old infants at high familial risk of autism

**DOI:** 10.1111/ejn.13751

**Published:** 2017-11-22

**Authors:** Ricarda Braukmann, Sarah Lloyd‐Fox, Anna Blasi, Mark H. Johnson, Harold Bekkering, Jan K. Buitelaar, Sabine Hunnius

**Affiliations:** ^1^ Department of Cognitive Neuroscience Donders Institute for Brain, Cognition and Behaviour Radboud University Medical Centre Nijmegen The Netherlands; ^2^ Donders Institute for Brain, Cognition and Behaviour Radboud University Nijmegen P.O. Box 9104 6500 HE Nijmegen The Netherlands; ^3^ Centre for Brain and Cognitive Development Birkbeck, University of London Cambridge UK; ^4^ Department of Psychology University of Cambridge Cambridge UK; ^5^ Karakter Child and Adolescent Psychiatry University Centre Nijmegen The Netherlands

**Keywords:** autism spectrum disorder, functional near‐infrared spectroscopy, infant, social stimuli

## Abstract

The social and communicative difficulties that characterize autism spectrum disorder (ASD) are considered the most striking feature of the disorder. Research has reported that individuals with ASD show abnormalities in the brain regions associated with the processing of social information. Importantly, a recent study using functional near‐infrared spectroscopy (fNIRS) found the first evidence of atypicalities in the neural processing of social information in 4‐ to 6‐month‐old infants at high familial risk of ASD. These findings provide an important step in the search for early markers of ASD and highlight the potential for neuroimaging techniques to detect atypical patterns of neural activity prior to the manifestation of most behavioural symptoms. This study aimed to extend the findings of reduced neural sensitivity to social stimuli in an independent cohort. Twenty‐nine 5‐month‐old infants (13 low‐risk infants, 16 high‐risk infants) were presented with social and non‐social visual stimuli, similar to the previous experiment. Importantly, a non‐social dynamic motion control condition was introduced allowing the comparison between social dynamic and non‐social, static, as well as dynamic stimuli. We found that while low‐risk infants showed activation to social stimuli in the right posterior temporal cortex, this activation was reduced in infants at high risk of ASD. Although the current sample size was relatively small, our results replicate and extend previous work and provide evidence for a social processing difference in infants at risk of autism. Future research will determine whether these differences relate to an eventual ASD diagnosis or may rather reflect the broader autism phenotype.

## Introduction

The social and communication difficulties that characterize autism spectrum disorders (ASDs) are considered the most striking feature of the disorder. Many researchers have studied the atypical patterns of behaviour related to understanding others’ minds, goals and intentions that can be observed in individuals with ASD (Yirmiya *et al*., 1998; Baron‐Cohen, 2000a; Brent *et al*., 2004; Boria *et al*., 2009; Zalla *et al*., 2010; Sparaci *et al*., 2014; Peterson *et al*., 2016). In typically developing adults, the processing of social information is associated with specific brain regions including areas in the temporal lobe – in particular the superior temporal sulcus (STS), temporoparietal junction (TPJ), orbitofrontal cortex, fusiform face area (FFA), as well as the amygdala (Adolphs, 2003). Several neuroimaging studies have reported that individuals with ASD show atypical responses to the processing of social information in these social brain regions (Baron‐Cohen *et al*., 2000b; Amaral *et al*., 2003; Zilbovicius *et al*., 2006; Pelphrey *et al*., 2011; Misra, 2014). Atypicalities include, for example, diminished responses to social sounds (Gervais *et al*., 2004) and faces (Jemel *et al*., 2006) as well as altered processing of biological motion (Kaiser & Pelphrey, 2012).

Cortical activation selective for social stimuli begins to develop early in infancy. Functional near‐infrared spectroscopy (fNIRS) is one of the neuroimaging techniques best suited to study cortical activation in young infants (Lloyd‐Fox *et al*., 2010, 2014). In fNIRS, measurements of change in absorption of near‐infrared light from sensors placed on the infant's head are used to infer cortical oxy‐ (HbO_2_) and deoxy‐haemoglobin (HHb) concentration changes associated with neuronal activation in the underlying tissue (Ferrari *et al*., 2004). Using fNIRS, Lloyd‐Fox *et al*. (2009) were the first to show that – similar to adults – visual social stimuli elicit activation in the posterior temporal cortex in infants by 5 months of age. In the following years, other studies followed reporting similar early cortical selectivity to social stimuli, such as vocal sounds or social gaze cues (Johnson *et al*., 2005; Grossmann *et al*., 2010, 2013; Blasi *et al*., 2011; Lloyd‐Fox *et al*., 2011b; Farroni *et al*., 2013). Given that neural tuning towards social stimuli is already present at such a young age, atypical information processing within social brain regions in ASD may also be visible early in infancy and could serve as a potential early marker of the disorder.

One way to study early social processing deficits in ASD is by means of prospective longitudinal studies. Siblings of children diagnosed with ASD have an increased risk of receiving a diagnosis themselves (ranging from 10 to 20%, compared to 1% in the general population, Constantino *et al*., 2010; Ozonoff *et al*., 2011). Following those infants during early development hence provides a unique opportunity to directly assess early markers of the disorder that may aid early detection, diagnosis and eventual treatment of ASD (Elsabbagh & Johnson, 2010; Bölte *et al*., 2013). Using such a prospective study design, Lloyd‐Fox *et al*. (2013) investigated the processing of complex dynamic social stimuli in 5‐month‐old infants at risk of ASD. In their experiment, infants were presented with engaging social videos of a female actor which were compared to a baseline of full‐colour static non‐social images. In addition, infants were also presented with auditory vocal and non‐vocal stimuli to assess temporal cortex responses to auditory stimuli. While typically developing infants showed specific activation in posterior temporal regions to the social compared to the non‐social stimuli, this activity was reduced in the high‐risk infants. Group differences were visible for both modalities, suggesting a generic difference in social information processing in the temporal cortex of infants at risk. In line with these findings, other studies have also reported differences in neural processing during the first year of life in high‐risk infants (Elsabbagh *et al*., 2009; McCleery *et al*., 2009; Bosl *et al*., 2011; Luyster *et al*., 2011; Wolff *et al*., 2012). Most behavioural atypicalities, on the other hand, become manifested gradually between the end of the first and the second year of life and are often subtle in nature (Elsabbagh & Johnson, 2010; Jones *et al*., 2014; but see Di Giorgio *et al*., 2016). Neuroimaging methods hence play an important role in the detection of early neural markers that precede the onset of behaviourally expressed symptoms.

The recent findings of atypical social processing in infants at risk of ASD by Lloyd‐Fox *et al*. (2013) are promising, yet data were collected from a relatively small number of participants (18 high‐risk and 16 low‐risk infants) and findings thus require replication. This study aimed to extend the previous findings of reduced neural sensitivity in high‐risk infants (Lloyd‐Fox *et al*., 2013) in an independent infant cohort. Functional NIRS data were collected from 5‐month‐old infants at high and low familial risk of ASD who were presented with social dynamic and non‐social static visual stimuli. In addition, our current experimental design was extended to include a dynamic non‐social control condition (similar to that used in a previous study of typically developing infants; Lloyd‐Fox *et al*., 2009). This condition was added to assess group differences in processing of social stimuli controlling for the amount of motion in the stimulus display. Although the spatial resolution of fNIRS is much better than that of electrophysiological measures (Lloyd‐Fox *et al*., 2010), it is difficult to determine the exact anatomical location from which the measured signal originates. As motion‐sensitive areas such as MT/V5 are located close to the posterior STS, differences in motion information between stimuli may result in a potential confound: Increased cortical activity may represent sensitivity to motion (MT/V5) rather than the processing of social aspects of the stimuli (STS) (Lloyd‐Fox *et al*., 2009). The previous study of infants at familial risk of ASD contrasted *dynamic* social stimuli with *static* non‐social stimuli (Lloyd‐Fox *et al*., 2013) and did not include a motion control condition. Therefore, while we believe from previous research in typically developing infants (see Lloyd‐Fox *et al*., 2009) that the channels identified in Lloyd‐Fox *et al*. (2013) were over the pSTS‐TPJ region for the low‐risk infants, we do not know whether stimulus motion could have had an impact on the observed response in the high‐risk infants. By contrasting social dynamic stimuli with non‐social dynamic stimuli, this study is able to assess differences between high‐ and low‐risk infants in the cortical processing of social information controlling for the amount of motion presented. Based on previous research (Lloyd‐Fox *et al*., 2013), we expected to find diminished neural hemodynamic responses in posterior temporal cortex to social stimuli in the high‐risk infants compared to low‐risk controls.

## Methods

### Participants

All infants who participated in this experiment were taking part in a longitudinal study on the early development of autism. High‐risk (HR) infants were included if they had at least one older sibling with a clinical diagnosis in the autism spectrum. For all children, a clinical report was available to the research team that was used to confirm the diagnosis of the older sibling. Low‐risk (LR) infants were included if they had at least one older typically developing sibling and no family history of autism. In addition, all infants had to be born full‐term (> 36 weeks) to be included. The study was approved by the local medical ethics committee (Commissie Mensgebonden Onderzoek (CMO) regio Arnhem – Nijmegen), and written informed consent was given by both parents prior to the start of the experiment.

Thirty‐five 5‐month‐old infants (16LR, 19HR) were enrolled and participated in the fNIRS experiment. Six infants (3HR) were later excluded due to poor data quality (3: 2HR), insufficient number of trials (1: LR) or experimenter error (2: 1HR). The final sample used for analysis hence consisted of 29 infants (13LR, 16HR, Table 1).

**Table 1:** Characteristics of the final sample

	N	Age	ELC standard score
Low risk	13 (4♀)	5.27(0.50)[4.63–6.01]	95.62(09.01)[74–110]
High risk	16 (9♀)	5.37(0.58)[4.70–6.51]	90.06(11.22)[61–106]
Total	29 (13♀)	5.34(0.54)[4.63–6.51]	93.55(10.49)[61–110]

Age and the MSEL Early Learning Composite (ELC) standard score are mean values with standard deviations reported in the parentheses. The range for the age and the ELC scores is reported in the square brackets. There were no significant differences between high‐ and low‐risk infants in age (*t*(27) = −0.07, *P* = 0.55), gender (*X* ^2^(1, *N* = 29) = 1.88, *P* = 0.17) or ELC standard scores (*t*(27) = 1.44, *P* = 0.16).

In addition to the fNIRS experiment described below, the development of each infant was assessed using the Mullen Scales of Early Learning (MSEL, Akshoomoff, 2006; Mullen, 1995). The MSEL is a standardized measure consisting of five scales [visual reception (VR), expressive (EL) and receptive language (RL), and gross (GM) and fine motor (FM)] which combined lead to the Early Learning Composite (ELC) standard score reflecting the overall development of the child. Importantly, we found no differences between the two infant groups for the overall ELC score (*t*(27) = 1.44, *P *=* *0.16) as well as for any of the five distinct subscales (GM: *t*(27) = 0.95, *P *=* *0.35; VR: *t*(27) = 0.96, *P *=* *0.35, FM: *t*(27) = 1.39, *P *=* *0.18, RL: *t*(27) = 1.28, *P *=* *0.21, EL: *t*(27) = −0.55, *P *=* *0.58), suggesting that our groups matched in their overall as well as domain‐specific development.

### Stimulus material

**Figure 1:**
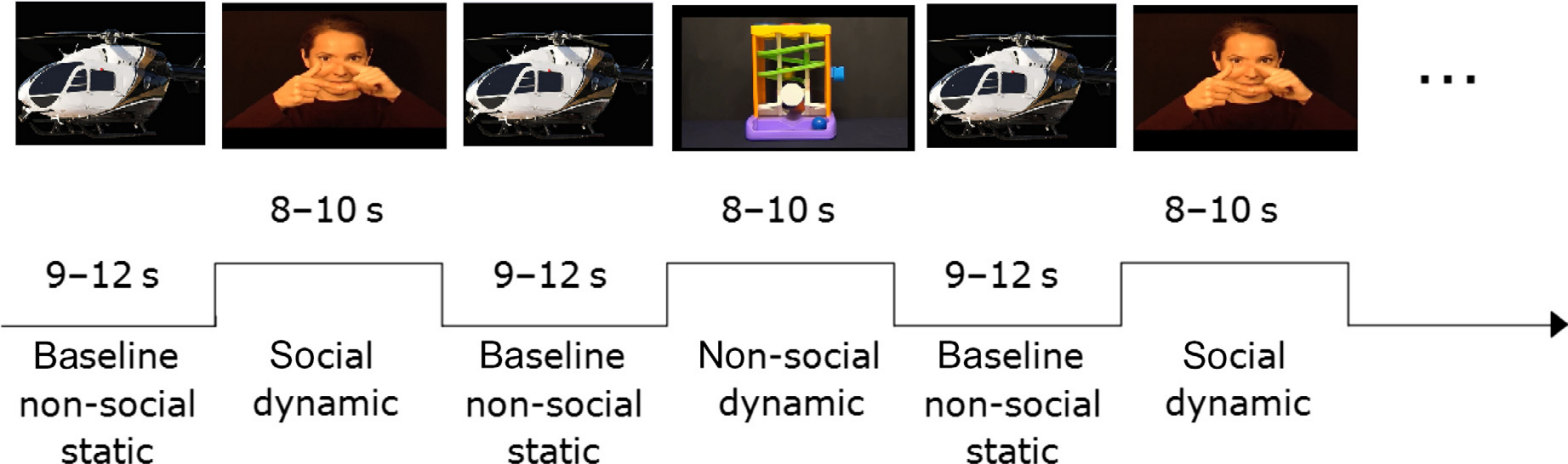
Timeline of the experimental blocks. Infants were presented with blocks of social and non‐social dynamic stimuli interspersed with a static non‐social baseline block. The experiment always started with a baseline block, but whether the first dynamic block was social or non‐social was counterbalanced between participants.

The stimulus material consisted of social and non‐social (dynamic) video stimuli as well as non‐social static images. The non‐social static baseline images were the same stimulus material as used by Lloyd‐Fox *et al*. (2013) and consisted of pictures of transportation devices (such as cars or helicopters). There were 19 different baseline stimulus images in total. Per baseline block, a random selection of six pictures was presented for a variable duration of 1–3 s per image. The social dynamic video stimuli were also based on Lloyd‐Fox *et al*. (2013), and the current social dynamic condition was the same as the visual social condition of the previous study. The social videos showed a combination of actions or movements performed by a female actor. These included for instance the actor moving her eyes from one side to the other, moving her mouth or performing hand games such as ‘peek‐a‐boo’ (Fig. 1). There were 6 different social dynamic videos in total. Per block, one of those videos was presented to the infant. The video presentation sequence was pseudorandomized, ensuring that each of the six videos had been presented once before a video was repeated. The non‐social stimuli consisted of videos from previous studies (Lloyd‐Fox *et al*., 2009) as well as newly created stimulus materials. Videos were selected to be as similar as possible to the social stimulus videos in the amount of presented motion. The final selection of the non‐social video stimulus set was based on a visual inspection of the motion patterns of the distinct social and non‐social videos. Specifically, the amount of motion was estimated for each stimulus video by looking at frame‐by‐frame changes in the summed squared differences in red, green and blue colour channels of all pixels (cf. Schippers *et al*., 2010; Meyer *et al*., 2015). Consecutively, the motion energy was compared between the social and non‐social stimuli and a selection of non‐social videos was made for inclusion in the current study. The non‐social dynamic videos showed a combination of moving toys or objects. These included, for instance, moving machines, spinning toys or toys that contained moving balls (Fig. 1). Similar to the social condition, the stimulus set of the non‐social condition consisted of a total of 6 different videos and one video was pseudorandomly presented during each of the non‐social dynamic blocks.

### Procedure

Infants were seated in a baby carrier on their parent's lap in a sound‐proof testing booth in front of a computer screen monitor (24 inch, 16:9, 1920 × 1080 pixels). After the infants were capped with the NIRS headgear, they were presented with blocks of dynamic video clips in alternation with a non‐social static baseline period (Fig. 1). Stimulus presentation was realized using matalb (Mathworks, Inc., Natick, MA; http://mathworks.com, Version 2011b), and infants’ behaviour was monitored online and videotaped for offline coding. If the participant disengaged from the display, the experimenter could play an attention getting sound to redirect the infant's attention back to the screen and the experiment continued until the infant was bored or showed signs of discomfort.

### NIRS data acquisition

**Figure 2:**
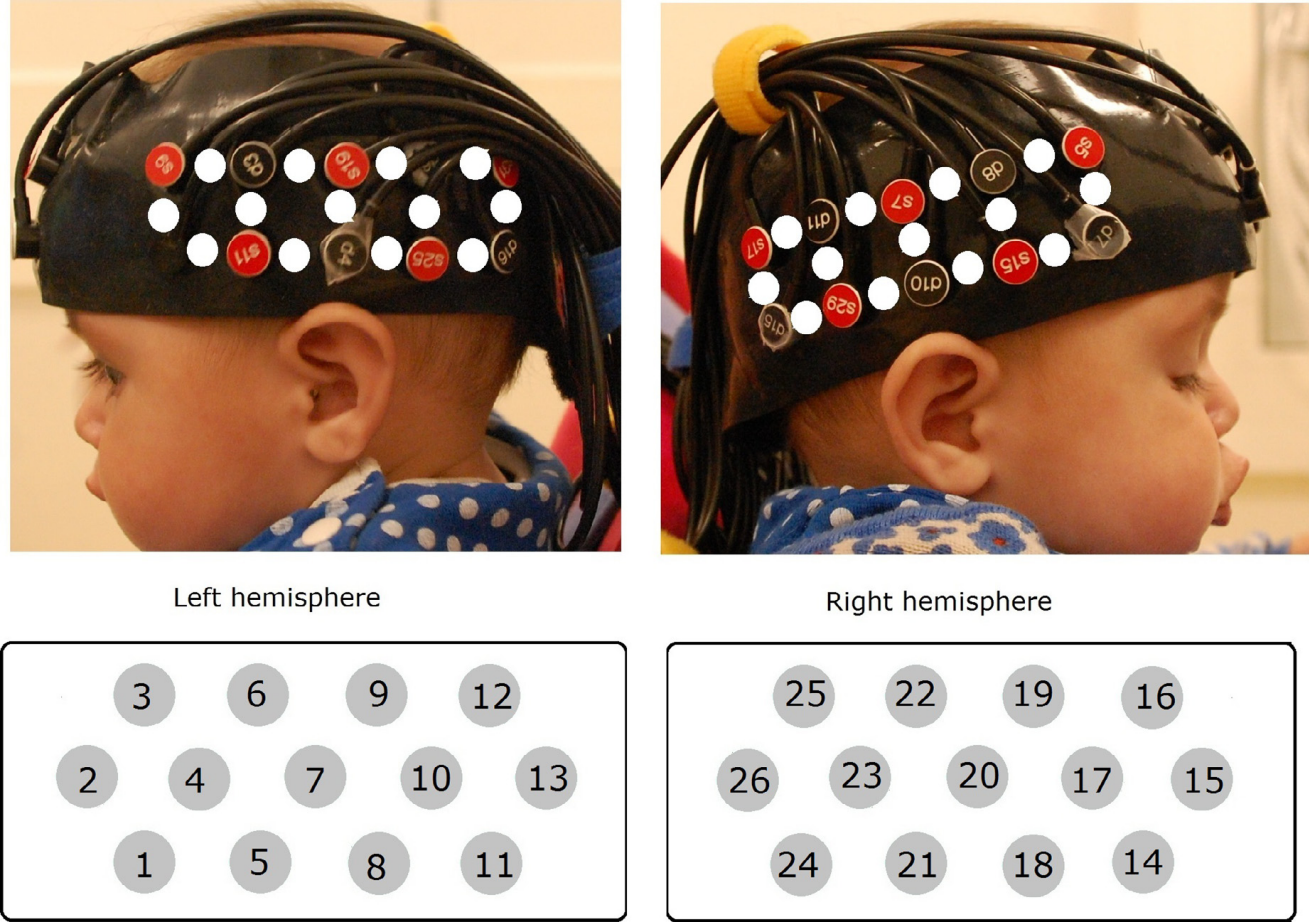
Head gear and channel location. The upper panels show an infant wearing the NIRS headgear. The white dots represent the approximate channel locations between each source–detector pair. The lower panels show a schematic of the recording channels and identify channel numbers. The infant's parents gave permission for this image to be published.

Near‐infrared spectroscopy data were collected using the University College London (UCL) topography system (Everdell *et al*., 2005) that emits near‐infrared light at two wavelengths (780 and 850 nm). A custom‐built headgear similar to Lloyd‐Fox *et al*. (2013) was used to collect data from 26 channels (10 detector and 10 source optodes) which were placed over the temporal cortex at an inter‐optode distance of 2 cm (Fig. 2). The headgear positioning was based on external anatomical landmarks of the infant's head, and placement was done such that the posterior area of the headgear approximately covered the STS‐TPJ area according to previous research, and a NIRS‐MRI co‐registration map of scalp location to anatomy developed for this age range (Lloyd‐Fox *et al*., 2009, 2013, 2014).

### Data pre‐processing

Infants’ looking behaviour was coded offline to ensure that trials were only included in the analysis if infants had watched at least 60% of the dynamic stimuli as well as 30% of the pre‐ and post‐stimuli static baseline (Lloyd‐Fox *et al*., 2013). Importantly, we verified that there were no differences in looking time for the two conditions and between low‐ and high‐risk infants (see Appendix S1).

The collected NIR attenuation data from each channel were assessed using artefact detection algorithms (Lloyd‐Fox *et al*., 2009, 2011a, 2013) implemented in matlab (Mathworks, Inc., Natick, MA; http://mathworks.com, Version 2015b). In line with previous work, channels were excluded if the coefficient of variation of the attenuation exceeded 10% or if the normalized power was larger than 35% with respect to the total power (Lloyd‐Fox *et al*., 2013). If an infant showed artefacts on more than half of the channels, the infant was excluded from further analysis.

Consecutively, the attenuation data were low‐pass filtered at 1.2 Hz and blocks of 22 s were extracted for each of the dynamic trials, consisting of the last 4 s of the static baseline trial, the dynamic trial (8–10 s) and the following static baseline trial (9–12 s). Linear trends within each block were removed by subtracting a line defined between the first and last 4 s of each block. Next, the data were transformed to HbO_2_ and HHb concentration changes using the modified Beer–Lambert law (Delpy *et al*., 1988) with a differential pathlength factor of 5.13 for infants (Duncan *et al*., 1995). Finally, a trial was rejected within a channel if HbO_2_ concentrations exceeded 3.5 μm during baseline or 8 μm during the dynamic stimuli. For a channel to be included in the statistical analysis for a particular infant, at least three valid artefact‐free trials were required. The number of infants that were included for a particular channel was hence variable.

### Data analysis

Data analysis followed closely the analysis steps from previous studies using a similar paradigm (Lloyd‐Fox *et al*., 2009, 2013). Hence, HbO_2_ and HHb concentration changes during the dynamic video presentation were assessed within a four‐second time window (10–14 s). This window was chosen based on other recent fNIRS studies (Lloyd‐Fox *et al*., 2013; Lloyd‐Fox *et al.,* 2017; Lloyd‐Fox *et al*., 2018) and taking into account that the hemodynamic response takes time to reach its peak after stimulus onset. We chose a slightly narrower time window in comparison with previous studies (e.g. Lloyd‐Fox *et al*., 2011b; Grossmann *et al*., 2013) as recent work by Lloyd‐Fox *et al*. (2017) has shown that such a narrow window around the peak of the response provides a more robust marker of activation. Using this window, we extracted concentration changes for both HbO_2_ and HHb. Although HBO_2_ has a higher signal‐to‐noise ratio and responses are often more consistent in infants, it is recommended to report activation changes for both HbO_2_ and HHb to provide a complete picture of cortical activation changes and to aid comparability between studies (Lloyd‐Fox *et al*., 2010; Tachtsidis & Scholkmann, 2016) and we followed these recommendations in the current study.

In a first analysis step, the averaged HbO_2_ and HHb concentration changes for the two dynamic conditions were compared to baseline using one‐sample *t*‐tests. To control for multiple comparisons, *P*‐values were corrected for the number of investigated channels using false discovery rate (FDR) methods (Benjamini & Yekutieli, 2001). Channels that showed significant activation from baseline were then further investigated. Importantly, activation was considered valid if channels showed an increase in HbO_2_ and/or a decrease in HHb. Channels for which HbO_2_ and HHb were significantly increasing or decreasing in unison were not included in the analysis, as the signal was then considered inconsistent with the usually elicited cortical response (see Lloyd‐Fox *et al*., 2013). For the channels that showed significant signal change from baseline, the peak change within the four‐second time window was then extracted for the dynamic conditions for further comparisons. In a first step, differences between the social dynamic and non‐social dynamic stimuli were assessed within the two infant groups using paired sample *t*‐tests. Then, in a final step, group differences in response to the dynamic stimuli were assessed using independent sample *t*‐tests.

## Results

On average, infants watched 8.86 social dynamic blocks (range 3–17), 8.86 non‐social dynamic blocks (range 3–17) and 16.55 static baseline blocks (range 5–35) and there was no difference in the amount of trials watched between the high‐risk and low‐risk infants (social: *t*(27) = 0.57, *P *=* *0.58; non‐social: *t*(27) = 0.37, *P *=* *0.72; baseline: *t*(27) = 0.91, *P *=* *0.37, for a more detailed report on the infants’ visual attention see Appendix S1). The mean number of infants included in the final analysis per channel was 27 (12LR, 15HR) ranging from 25 (12LR, 13HR) to 28 (12LR, 16HR).

**Figure 3:**
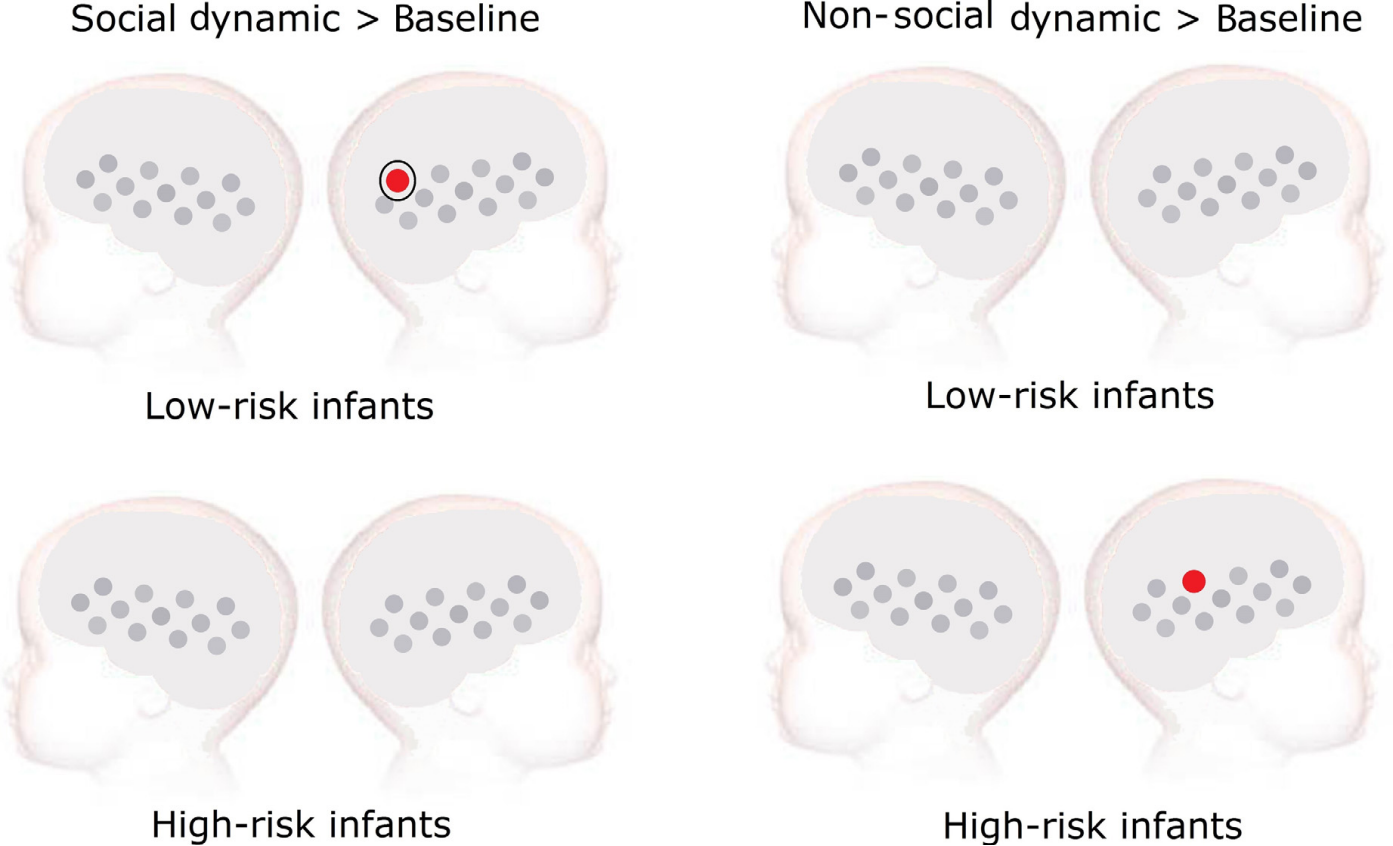
Results of the analysis comparing cortical activation to dynamic social (left) and non‐social stimuli (right) with respect to the non‐social static baseline. Low‐risk infants (upper panels) showed increased HbO_2_ concentration changes for the social dynamic stimuli in channel 25. High‐risk infants (lower panels) showed increased HbO_2_ concentration changes for the non‐social dynamic stimuli in channel 22. Significant group differences were found in channel 25, indicated by the black circle.

**Figure 4:**
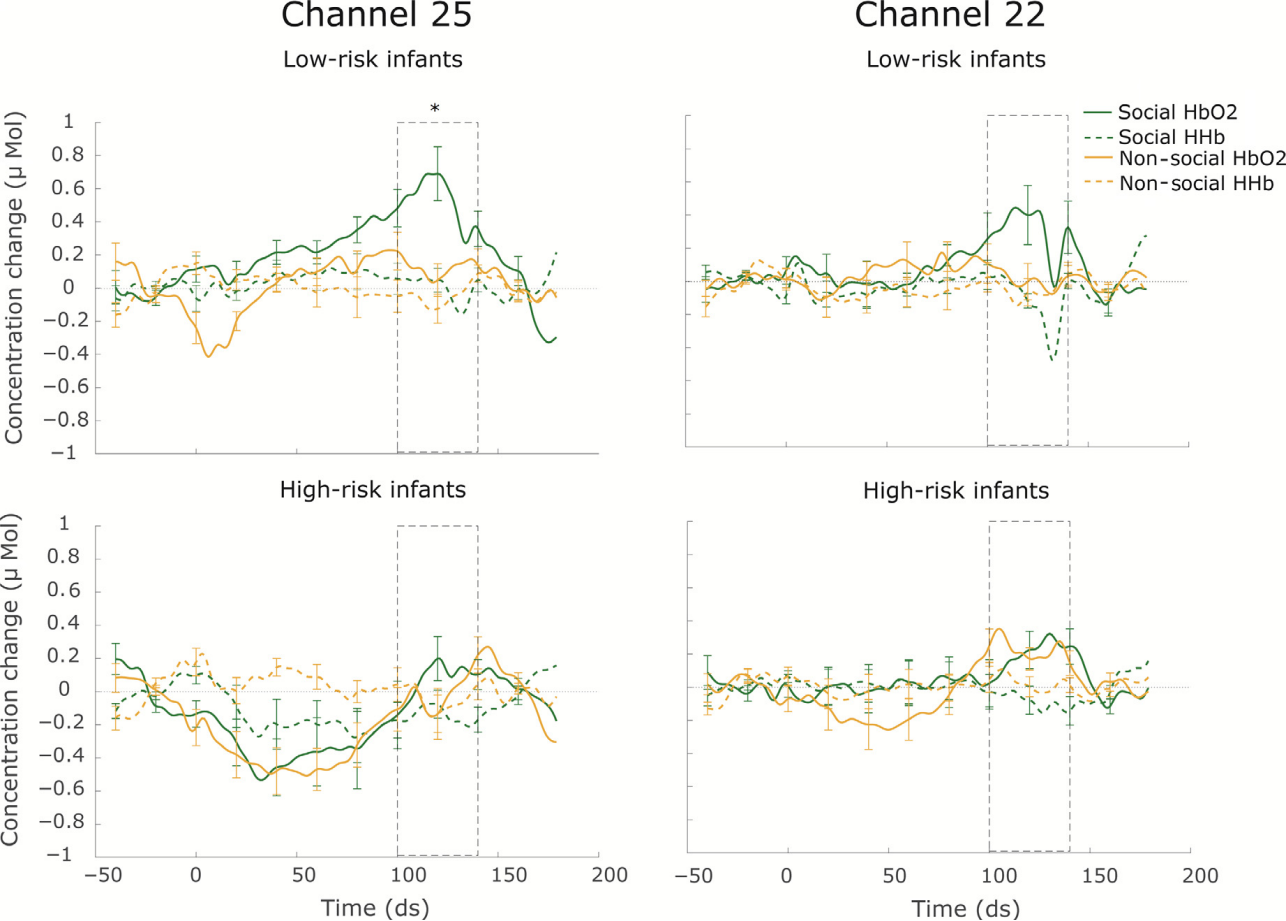
Averaged Haemoglobin concentration changes for the social and non‐social dynamic condition for the low‐risk (LR, upper panels) and high‐risk (HR, lower panels) infants in channel 25 (left) and 22 (right). Time point 0 represents the onset of the dynamic video block, and the dotted window shows the time window on which the statistical comparisons are based. Group differences are indicated by the asterisk.

Cortical activation to the dynamic stimuli was assessed with respect to the non‐social static baseline period. The results reported as significant in the following section have been corrected for multiple comparisons using false discovery rate (FDR) methods. An overview of the significant channels based on the uncorrected *P*‐values can be found in the Table S1. An overview of the results is shown in Fig. 3. For the low‐risk infants, the analysis revealed a significant increase in HbO_2_ for the social dynamic condition in channel 25 (*t*(11) = 4.07, *P* = 0.048, corrected using FDR), which was positioned over the right pSTS‐TPJ region. No significant activation was found for HbO_2_ for the non‐social dynamic condition (*P* > 0.46 for all tests, corrected using FDR). No significant activation was found for HHb for either condition (*P *>* *0.12 for all tests, corrected using FDR). For the high‐risk infants, no channels showed significant HbO_2_ activation for the social dynamic condition (*P *>* *0.27 for all tests, corrected using FDR). There was, however, a significant increase in HbO_2_ concentration changes for the non‐social dynamic condition in channel 22 (*t*(15) = 3.92, *P *=* *0.040, corrected using FDR), which was positioned over the same right pSTS‐TPJ region. No significant activation was found for HHb for either condition (*P *>* *0.33 for all tests, corrected using FDR). Channels 22 and 25 were hence selected for further analysis of condition and group differences in HbO_2_ concentration changes. Figure 4 shows the averaged HbO_2_ and HHb time courses for the social and non‐social dynamic condition in those channels for the low‐ and high‐risk infants. Condition differences were significant for the low‐risk infants in channel 25, as the social dynamic stimuli elicited significantly larger HbO_2_ concentration changes than the non‐social dynamic stimuli (*t*(11) = 2.82, *P *=* *0.02, Cohen's *d *=* *0.94). There were no condition differences in channel 22 (*t*(10) = 1.47, *P *=* *0.17) for the low‐risk infants. Importantly, for the high‐risk infants, no condition differences were found for either channel (22: *t*(14) = −0.67, *P *=* *0.51; 25: *t*(15) = 0.34, *P *=* *0.74). In a last step, group differences were assessed and independent sample *t*‐tests showed that the HbO_2_ response to social dynamic stimuli in channel 25 was significantly larger for the low‐risk infants compared to the high‐risk group (*t*(26) = −2.22, *P *=* *0.04, Cohen's *d *=* *0.87, Fig. 3). There were no group differences for the non‐social dynamic stimuli in channel 25 (*t*(26) = −0.09, *P *=* *0.93), and no group differences were found for channel 22 for either condition (social: *t*(24) = −0.57, *P *=* *0.57, non‐social: *t*(24) = −0.67, *P *=* *0.51).

### Differences in cortical responses between channel 22 and channel 25

As both channels 22 and 25 are located over the pSTS‐TPJ region, we would have expected similar patterns of results for these two channels, rather than the group and condition‐specific difference reported above. To investigate whether patterns of activation were indeed different for the two channels, we conducted an additional post hoc analysis comparing activation in channel 22 and channel 25. A 2 × 2 repeated measures anova was performed for each of the infant groups separately, using channel (Ch22, Ch25) and condition (social, non‐social) as within subject factors. For the low‐risk group, we found a main effect of condition (*F*_1,10_ = 10.46, *P *<* *0.01), indicating that HBO_2_ responses for the social condition were overall larger than responses in the non‐social condition. The main effect of channel (*F*_1,10_ = 2.31, *P *=* *0.16) and the interaction effect were not significant (*F*_1,10_ = 0.50, *P *=* *0.50) for the low‐risk infants. For the high‐risk group, there was no significant main effect of condition (*F*_1,14_ = 1.27, *P *=* *0.28) or channel (*F*_1,14_ = 2.56, *P *=* *0.13), and the interaction effect did not reach significance either (*F*_1,14_ = 2.53, *P *=* *0.13). These results suggest that for both infants, the two channels did not differ significantly from each other. Moreover, the finding that a main effect of condition is present for the low‐risk group but not for the high‐risk group is in line with the results reported in the main analysis showing increased posterior temporal cortex activation for social compared to non‐social dynamic stimuli in the low‐risk controls only.

### Initial decrease in activation in the high‐risk infants

In the time courses displayed in Fig. 4, an initial decrease in the HbO_2_ response for both the social and non‐social conditions seems to be visible in channel 25 for the high‐risk infants. To investigate this response further, we performed an additional post hoc analysis testing for activation from baseline using a 3–7 s time window surrounding the minimum of the response visible for the high‐risk infants in Fig. 4. This analysis revealed that both dynamic conditions indeed elicited a significant decrease in HbO_2_ in channel 25 for the high‐risk infants (social: *t*(15) = −2.18, *P *=* *0.046, uncorrected; non‐social: *t*(15) = −3.57, *P *=* *0.003, uncorrected). No significant differences from baseline were found for HHb (social: *t*(15) = −1.37, *P *=* *0.191, uncorrected; non‐social: *t*(15) = 1.91, *P *=* *0.075, uncorrected).

## Discussion

Previous research found that 5‐month‐old infants at high risk of developing autism spectrum disorders show reduced neural sensitivity to social stimuli (Lloyd‐Fox *et al*., 2013). In the present study, a similar experimental design was implemented and our results extend the original findings. In line with our hypothesis, low‐risk infants showed significant activation over right posterior temporal cortex in response to social stimuli, whereas this response was not significant in the high‐risk infants. Importantly, we compared social and non‐social dynamic stimuli which were similar in the amount of motion in the stimulus display. This contrast enabled us to assess whether activation of the posterior temporal cortex was modulated by the social aspects rather than representing activation originating from motion‐sensitive brain regions. Confirming our hypothesis, low‐risk infants showed a socially selective cortical response in the right posterior temporal cortex: HbO_2_ concentration changes were larger in response to the social dynamic than the non‐social dynamic condition, suggesting that the reported activation indeed originates from regions involved in the processing of social information (i.e. pSTS‐TPJ, see also Lloyd‐Fox *et al*., 2009). In contrast, social dynamic stimuli did not elicit any significant cortical activation in the high‐risk infants. Rather, we found significant activation for the non‐social condition in the right posterior temporal cortex with respect to baseline for this infant group. Similar activation from baseline to non‐social dynamic stimuli has also been reported previously in typically developing infants (Lloyd‐Fox *et al*., 2009) and is likely due to the more engaging nature of the dynamic stimuli compared to the static baseline. Although it is interesting that the activation to the non‐social condition in the high‐risk infants did survive FDR correction, whereas the social activation did not, it is important to note that we did not find significant differences between the social and non‐social stimuli. Rather, the time courses of the HbO_2_ responses shown in Fig. 4 appeared very similar for both conditions. These results suggest that both conditions were processed similarly by the high‐risk infants and that the socially selective processing visible in the low‐risk infants was diminished in the at‐risk group.

Our findings complement previous studies reporting early social processing differences in at‐risk infants compared to typically developing controls (Elsabbagh *et al*., 2012; Lloyd‐Fox *et al*., 2013; Jones *et al*., 2016). Moreover, our results are in line with previous work in older individuals with ASD showing atypical social processing (Gervais *et al*., 2004; Jemel *et al*., 2006) and attentional orienting (Klin *et al*., 2009), as well as difficulties in the integration of complex dynamic social information (Shah *et al*., 2016). It has been theorized that the atypicalities in social processing observed in ASD may be the result of an early failure to orient towards social information (Jones *et al*., 2008; Johnson, 2014). Typically developing infants show an early bias drawing their attention towards socially relevant stimuli, like faces (Morton & Johnson, 1991; Farroni *et al*., 2002; Johnson, 2005). The interactive specialization theory postulates that the cortical social brain network emerges through an interaction of those early attentional biases and environmental experiences. Abnormalities in the bias to orient towards socially relevant stimuli in ASD may hence lead to a cascade, disrupting typical developmental processes (Johnson, 2001, 2011). There is an ongoing debate about this hypothesis as several studies have recently reported typical patterns of attention to social stimuli in young infants at‐risk of ASD (Elsabbagh *et al*., 2013; Jones & Klin, 2013). Jones & Klin (2013), for instance, showed that fixations to the eye region during the presentation of faces were similar for 2‐month‐old high‐risk infants who later receive a diagnosis and low‐risk controls. The researchers reported that differences in looking at the eyes of others only emerged later, between 2 and 6 months of age. In contrast, Di Giorgio *et al*. (2016) recently reported differences in attention to social stimuli already in newborns at high risk of ASD. Future studies will need to integrate the different findings and provide more detailed reports of the development of social processing in infants at risk of ASD. Our current findings, showing that by 5 months of age socially selective cortical activation is diminished in high‐risk infants, suggest that atypicalities in social processing are present during the first half year of development, but more research will be needed to assess when these deviations first emerge.

One difference between the current study and the previous findings by Lloyd‐Fox *et al*. (2013) that needs consideration is the extent of cortical activation we observed. While previously, broader bilateral temporal cortex activation to social stimuli was found (Lloyd‐Fox *et al*., 2009, 2013), significant activation was limited to right posterior temporal cortex in the current experiment. We argue that this difference can be explained by the stricter measures that we applied to control for multiple comparisons. Whereas the previous study reported uncorrected *P*‐values, our results were corrected using a false discovery rate approach which reduced the number of channels that were considered significant (see Table S1 for a complete report of all significant corrected and uncorrected *P*‐values). In addition, the sample size of the current experiment was slightly lower than in the previous study which may have led to less power for detecting cortical activation.

An observation we would like to further discuss is the apparent initial decrease in the HbO_2_ response for both the social and non‐social conditions visible in channel 25 in the high‐risk infants (see Fig. 4). Post hoc additional analyses of this initial response revealed that the HbO_2_ decreased significantly for both the social and the non‐social condition, whereas no significant differences were apparent for HHb. As the two chromophores were thus not decreasing in unison or mimicking each other, we would not consider this response to represent an artefact. Rather, we would argue that the observed initial deactivation in the high‐risk infants may represent a meaningful characteristic of the hemodynamic response to the presented social and non‐social stimuli. Interestingly, Lloyd‐Fox *et al*. (2018) found a similar pattern of early decreased activation in a group of high‐risk infants that went on to develop ASD in toddlerhood, supporting the notion that this phenomenon may be a relevant characteristic of early autism. However, deactivation of HbO_2_ is difficult to interpret and it remains unclear what is driving this response. Therefore, additional research using larger samples is needed to replicate this finding and further investigate its underlying physiology and significance.

Following multiple comparison correction, our results showed significant activation for the low‐risk infants for the social dynamic stimuli in channel 25 only and for the high‐risk infants for the non‐social dynamic stimuli in channel 22 only. While these findings could reflect disparate patterns of activation for these two channels, post hoc analyses showed no significant main effect of channel or any interaction between channel and condition for neither the low‐ nor the high‐risk infants. These results suggest that overall there was no substantial difference in response to channels 22 and 25. The observed differences in significant results for the two channels from the main analyses may therefore have been influenced by the relatively low sample size and the strict multiple correction criteria that was applied, rather than reflecting disparate patterns of activation. Importantly, the post hoc analysis did show a significant main effect of condition for the low‐risk group, but not for the high‐risk group, in line with the results from the main analysis.

Despite a slightly smaller sample size and more conservative analysis approach, we replicated the expected pattern of increased cortical activation within the pSTS‐TPJ region to social stimuli in low‐risk controls which was absent in the high‐risk group. Our study thus illustrates that fNIRS is a powerful technique which is able to detect atypicalities in brain function during early infancy. While many of the behavioural red flags of developing ASD – such as lack of response to own name or difficulties in joint attention – start to emerge only around the end of the first or second year of life (Zwaigenbaum *et al*., 2005; Palomo *et al*., 2006; Jones *et al*., 2014), this study and other neuroimaging experiments have shown group differences earlier in development (Bosl *et al*., 2011; Wolff *et al*., 2012; Lloyd‐Fox *et al*., 2013). We currently have no information on whether the infants in our sample will develop typically or receive a diagnosis within the autism spectrum disorder at a later age. Therefore, while our results may be related to early autism, they may also indicate a risk group effect that is unrelated to a later ASD diagnosis. Likewise, some studies have shown that early group differences can be present in at‐risk infants (Merin *et al*., 2007) while not being related to a later ASD diagnosis (Young *et al*., 2009), while others have shown that early neural responses can be associated with later diagnoses of ASD (Elsabbagh *et al*., 2012; Lloyd‐Fox *et al.,* in press). Whether our current results represent an early marker of ASD or rather a characteristic of the risk group can be investigated once outcome data are available for our sample.

At the age of 36 months, a preliminary diagnosis of ASD can be made enabling researchers to classify high‐risk participants into groups of infants that do develop ASD (HR‐ASD) and those that do not (HR‐noASD). Recent findings from a collaborating laboratory using a similar fNIRS paradigm (Lloyd‐Fox *et al*., 2013) suggest that atypicalities in social processing may indeed be especially pronounced in high‐risk infants that receive a diagnosis of ASD at 36 months (Lloyd‐Fox *et al*., 2018). The researchers found that HR‐ASD infants showed diminished social brain network activation to visual and auditory social stimuli compared to low‐risk controls, providing the first evidence that these neural signatures may have the potential to be an early marker of the disorder. Their results were based on a small sample of 5 HR‐ASD infants, so it will be important to establish whether those infants from our sample who go on to receive a diagnosis of ASD at 36 months also show similar patterns of atypicality. Furthermore, in line with previous prospective infant ASD research (Kaiser *et al*., 2010; Elsabbagh *et al*., 2013), Lloyd‐Fox *et al*. (2018) found that differences between HR‐ASD and HRD‐noASD infants were not as strong as those with LR infants, suggesting that altered cortical responses to social stimuli may also be present in the broader autism phenotype (BAP). The BAP describes the finding that unaffected family members of individuals with autism share characteristics of the disorder at a subclinical level (BAP, Parr & Le Couteur, 2013; Piven *et al*., 1997). Getting a clearer picture of the characteristics of the BAP over development as well as of differences that can be predictive of ASD in the high‐risk infants will greatly benefit our understanding of the disorder and aid early detection and diagnosis. To enable those more detailed analyses in the future, data from larger samples will be required. Once the infants from the current study reach the age of 36 months, data can be pooled with other samples – such as the sample from Lloyd‐Fox *et al*. (2018) – to create a large data set for further analysis.

Taken together, our findings provide compelling evidence for an early social processing difference in 5‐month‐old infants at risk of ASD. Future research will determine whether these differences relate to an eventual diagnosis or may rather reflect the broader autism phenotype.

## Conflict of interest

The authors have no conflict of interest to declare.

## Author contributions

R.B. contributed to all aspects of this experiment: design, data collection and data analyses. S.L‐F & A.B. contributed to the design and data analyses. M.H.J., H.B., J.K.B. & S.H. contributed to the design. All authors (R.B., S.L‐F., A.B., M.H.J., H.B., J.K.B., S.H.) contributed to interpreting the results and to writing the manuscript.

## Data accessibility

Data are stored in the EUAIMS Data Repository, and requests for data should go through the EUAIMS and Eurosibs network data access policies. Please visit http://www.eurosibs.eu for contact details.

**Abbreviations**
(p)STS: (posterior) Superior temporal sulcus
ASD: autism spectrum disorder
BAP: broader autism phenotype
FDR: false discovery rate
FFA: fusiform face area
fNIRS: functional near‐infrared spectroscopy
HbO_2_: oxy‐haemoglobin
HHb: deoxy‐haemoglobin
HR: high‐risk
LR: low‐risk
TPJ: temporoparietal junction

## Supporting information

Table S1. Results from the one‐sample *t*‐tests assessing significant changes in HbO_2_ and HHb with respect to baseline in the low‐risk and high‐risk infant group.
*Appendix S1*. Analysis of group and conditional differences in stimulus attention.
