# Supplementary material for: Diminished socially selective neural processing in 5‐month‐old infants at high familial risk of autism
Source: Eur J Neurosci. 2017 Nov 22;47(6):720–8. doi: 10.1111/ejn.13751 (PMC5943701; doi:10.1111/ejn.13751)
Supplement: Supplementary file 1 [file EJN-47-720-s001.pdf]

EJN European Journal of Neuroscience FENS Federation of European Neuroscience Societies

Diminished socially selective neural processing in 5-month-old infants at high familial risk for autism

Ricarda Braukmann, Sarah Lloyd-Fox, Anna Blasi, Mark H. Johnson, Harold Bekkering, Jan K. Buitelaar & Sabine Hunnius

Review timeline:

Submission date:	19 December 2016
Editorial Decision:	05 February 2017
Revision received:	24 April 2017
Editorial Decision:	30 May 2017
Revision received:	25 June 2017
Accepted:	30 June 2017

Editor: Sophie Molholm

1st Editorial Decision 05 February 2017

Dear Ms. Braukmann,

We have now received both reviews of your manuscript. All agree that this is an important area of investigation and see the significance of this type of data, but they also raise concerns regarding the limitations of the study and question whether one can justifiably draw conclusions regarding risk biomarkers from these data. However, we understand that this is a difficult to acquire sample and that these data may provide unique insights, even if these must be considered with great caution given the limits of the approach and the size of the samples. It will be important that you make clear in the title and abstract, as well as elsewhere, the preliminary nature of these findings.

Obviously, a resubmission of your manuscript could only be considered on the basis of extensive revisions. When you prepare your re-submission, please consult the Author's Checklist provided at the bottom on this email, notably with regard to characterization of reagents and reporting of statistical analyzes in the Results section and Figure legends.

Thank you for submitting your work to EJN.

Kind regards,

John Foxe & Paul Bolam
co-Editors in Chief, EJN

Reviews:

Reviewer: 1 (Sasha Key, Vanderbilt Kennedy Center, USA)

Comments to the Author
The manuscript reports a replication and extension of the author's previous work aimed at identifying early neural markers of deficits in social information processing. Using fNIRS and social and nonsocial dynamic stimuli compared to the static nonsocial stimuli, the authors report diminished neural activation to social stimuli in 5-month-old sibs-ASD vs. low-risk typical infants. The findings contribute to the field of early social information processing and ASD, but in the present form, the manuscript also presents several concerns with the extent of the reported analysis and the strength of the proposed conclusions.

1. Since fNIRS is still relatively new, additional explanation of the analytic approach would help increase clarity:
- how the time windows of interest were determined?
- given the varied length of the individual stimuli, could a 22s analysis window correspond to somewhat different events across trials and conditions?
- why was the particular 4-sec window within the overall analysis segment used to extract the change

values? For example, the sibs-ASD group appears to show a sizeable and sustained reduction in HbO2 and HHb in response to the social stimuli prior to the analyzed window. Is there a functional interpretation for this observation or is it an artifact?
- could the individual differences of infants' attention to the stimuli (e.g., continuous vs. on/off attention within a trial) have an effect on the data?

2. The Data Analysis section stated that both HbO2 and HHb concentration changes were investigated, but the results do not mention any HHb results.

3. If channels 22 and 25 are positioned in spatial proximity and interpreted to reflect activity of the same pSTS-TPJ region, what would be the reason for their disparate results within and between the two subject groups?
Figure 4 suggests that changes in the HbO2 response to social stimuli could have been present at both channels, and the lack of statistical significance could be attributed to low power due to a small sample size. Similarly, while the results for sibs-ASD report an "increase in HbO2 concentration for the nonsocial dynamic condition in channel 22", it is not clearly visible in Figure 4.
It would be informative to report the effect sizes for the observed condition and group differences, and also to test whether there was a significant difference in signal between channels 25 and 22.

4. The discussion section overstates the results. While the sibs-ASD group did not show a statistically significant change in HbO2 for the social stimuli, Figure 4 argues against them not showing "any social-specific activation".
It is also surprising that the authors did not examine any associations between the fNIRS data and the behavioral measures of social-communicative functioning, as such analyses could help with the functional interpretation of the results. Given that the study sample comes from a larger longitudinal study, some relevant behavioral assessments are likely available.
In the absence of any brain-behavior association, the conclusion about "an early social processing deficit" in sibs-ASD is overly strong as not all brain differences translate into observable behavioral limitations, and most of the 16 sibs in this study may develop typically. Also, the discussion fails to consider the possibility of the observed fNIRS difference in the small sample at 5 months not being related to any later outcomes (e.g., Merin et al. (2007) found differences in gaze patterns in 6-month-old sibs-ASD, which later turned out not be predictive of ASD symptoms or severity as shown in Young et al. (2009)).

Minor issues:
How the presence/absence of the ASD diagnosis in the siblings was determined?
Was audio included with the visual stimuli?
Could the authors clarify why they opted to use multiple t-tests vs. a mixed model ANOVA?

Reviewer: 2 (James McPartland, Yale Child Study Center, USA)

Comments to the Author
This manuscript examines the processing of social stimuli in five-month-old infants at high risk or normal risk for autism. Measures of HbO2 and HHb were collected via fNIRS to assess the cortical activation in response to social and non-social dynamic stimuli and static non-social stimuli. The authors report significant differences in response to social dynamic stimuli compared to non-social dynamic stimuli for the low-risk infants, but no social-specific response for the high-risk infants. Additionally, they report significantly more activation in response to social dynamic stimuli for the low risk infants compared to the high risk infants. The authors interpret these findings as providing evidence for "an early social processing deficit in 5-month-old infants at risk for ASD". Strengths of this paper include the experimental design as well as using a False Discovery Rate (FDR) to correct for multiple statistical comparisons. While the paper does identify and attempt to correct for the confound of dynamic stimuli in the previous work, more stringent methodology could further control for this confound. Additionally, claims of a social processing deficit are not strongly supported by the experimental findings. These concerns are elaborated upon below.

Major concerns:
1. This paper did not sufficiently address the potential influence of differences in looking patterns or attention to stimuli between the HR and LR groups. The authors attempted to control for attention by ensuring that groups had comparable numbers of trials included/excluded based on a criteria of 60% attention. Please provide explanation and justification for this choice of criteria as well as how attention criteria were operationalized. Without this clarification, it is unclear whether there may have been differences in the distribution of attention throughout the trial. For example, depending on if the 60% attention threshold was met by looking at the beginning, the middle, the end, or randomly interspersed glances throughout the 8-10s trial, these looking pattern differences could account for group differences in brain activation. Please calculate percent of time looking and number of looks for both groups for each condition to identify group differences, condition differences, and interaction effects.

2. The conclusion that the findings provide compelling evidence for an early social processing deficit is not supported by the experimental findings. Rather, phrasing the findings as an early social processing difference or abnormality would be more appropriate, as deficit connotes an impairment that is not demonstrated. Given that the fNIRS headgear is restricted to frontal and temporal lobes of the brain, HR infants could be discriminating between social and non-social stimuli in brain regions that were not examined in the present study.

3. In the analysis section, the expectation of activation peak during 10-14 second time window is based on an in press paper. Please confirm that this time window is appropriate for both high risk and low risk infants in this sample.

4. Stimuli set:
a. Control condition doesn't appear adequate for purpose of the paper. Can two baselines work (i.e. static social and static non-social) to control for both social and non-social conditions? Or the static baseline could be more neutral such as a crosshair?
b. The amount of motion in the social and non-social stimuli is claimed to be matched. Please quantify the matching procedure.
c. Was luminance matched?
d. Overall, the description of the stimuli set was not clear. It's not clear whether each dynamic block is a single video or a combination of multiple counterbalanced videos. Figure 1 could be labeled with "baseline," "dynamic social," and "dynamic non-social" labels to clarify the presentation of stimuli. The number of different static and dynamic stimuli needs to be clarified.

5. Sample differences
a. Please examine potential differences between high risk and low risk groups in terms of sex distribution and discuss how this could influence interpretation of the results.
b. Please justify use of ELC as matching criteria. The inclusion of gross and fine motor seem less relevant; confirm that groups were specifically comparable on RL, EL, and VR.
c. Additionally, the nearly significant ($p=.09$) group difference on ELC raises a concern about cognitive differences accounting for the results for a sample of this size. Please confirm experimental effects exist independent of IQ differences.

Minor concerns:
1. In Table 1, please add ranges to age and ELC distributions.

2. The introduction could be tightened up by avoiding generalities like "many researchers have studied" or "many studies have reported". Most of the introduction and discussion sections are based off of previous research from the authors and do not deeply engage with what has previously been done in the literature.

3. Please discuss the use of both HbO2 and HHB in analysis for a general audience.

4. Clarify how sound was controlled for. If sound was used to regain the infants' attention these trials should have been excluded. Based on the author's previous paper, HR and LR groups may be responding differently to vocal and non-vocal stimuli, which could compromise the findings in the current paper.

5. Elaborate on the implications of the finding that HR infants have greater activation to non-social dynamic stimuli in relation to baseline.

6. Please justify the decision to further analyze channels 22 and 25 due to significantly greater activation during dynamic compared to static stimuli. Significantly greater activation in response to dynamic compared to static stimuli would suggest that the activation is in response to motion. This presents the confound with motion that the authors were intending to avoid.

7. In Figure 4, in channel 25 for high risk infants the HbO2/HHb isn't looking as expected. In previous work (Lloyd-Fox et al., 2013), the authors said "if HbO2 and HHb were either to increase or decrease significantly in unison, the signal was considered inconsistent with a haemodynamic response to functional activation, and not reported in analyses", but this looks like exactly what's happening in channel 25 for HR infants. This seems to call into question the findings of between-group differences, since those were only observed in channel 25.

Authors' Response 24 April 2017

Response to Reviewer 1
Major concerns:

1. Since fNIRS is still relatively new, additional explanation of the analytic approach would help increase clarity:

1.1 How the time windows of interest were determined?

The time window of interest was chosen based on other recent fNIRS studies and taking into account that the hemodynamic response takes time to reach its peak after stimulus onset (Lloyd-Fox et al., 2013, Lloyd-Fox et al., 2016; Lloyd-Fox et al., under review). While other previous studies have applied broader time windows (Grossmann, Lloyd-Fox, & Johnson, 2013;Lloyd-Fox, Blasi, Mercure, Elwell, & Johnson, 2011), we chose a narrower window of 10-14s, as recent work by Lloyd-Fox et al. (2016) has shown that such a narrow window around the peak of the response provides a more robust marker of the cortical activation elicited by the stimulus presentation.
The description of time window selection has been extended in the method section of the manuscript to clarify the rationale for choosing this specific time window (see: Methods - Data analysis, p. 11-12).
We would further like to refer to our answers to the reviewer's comment 1.3 (page 3) which discuss the choice and validity of the time window in more detail.

1.2 Given the varied length of the individual stimuli, could a 22s analysis window correspond to somewhat different events across trials and conditions?

The length of the different baseline and condition blocks indeed varied over trials, but this slight variation was similar to previous studies (Lloyd-Fox et al., 2009, 2013). Importantly, the 22s analysis window always started with 4 seconds of baseline followed by the social or non-social dynamic condition and the subsequent baseline. The onset of the dynamic condition within the analysis window was hence always the same for all trials, irrespective of the length of the individual blocks. There were also no differences in the average duration of the social and non-social dynamic condition as those durations were matched. We therefore expect no differences in the response across the trials or conditions related to the variable individual stimulus length.

1.3 *Why was the particular 4-sec window within the overall analysis segment used to extract the change values? For example, the sibs-ASD group appears to show a sizeable and sustained reduction in HbO2 and HHb in response to the social stimuli prior to the analyzed window. Is there a functional interpretation for this observation or is it an artifact?*

As mentioned in response to the reviewer's comment 1.1 (page 2), this specific time window of 10-14s was chosen based on previous findings which suggest that the hemodynamic response for a trial of this length peaks around this time after stimulus onset (Lloyd-Fox et al., 2013; Lloyd-Fox et al., under review) and that results are more robust when a narrow window of 4 seconds around the peak is applied (Lloyd-Fox et al., 2016). Based on the reviewer's comment, we reanalyzed our data using a 0-14s time window to assess whether our results depend on the use of this small window. Importantly, using a broader window, we still found a significant change from baseline for HbO_2 concentration changes to the social condition in the low-risk infants in channel 25 ($t(11)$=, $p<0.001$, FDR-corrected). Moreover and similar to the main analysis, we found a significant difference in this channel between the social and non-social condition in the low-risk infants ($t(11)=-3.09$, $p=0.01$) and between the low- and high-risk group for the social condition ($t(26)=-3.07$, $p=0.01$). These results are in line with the findings reported in the main manuscript and suggest that the conclusions of our paper are not merely a result of a narrow analysis window, but rather remain the same, even if a very broad time window is used. Importantly, and in line with a recent study by Lloyd-Fox et al. (2016), a narrower time window around the peak as used in the main analysis resulted in more significant channels, suggesting that it indeed increased the sensitivity to detect cortical activation. We have extended our description of the choice of analysis window in the methods section of the analysis (see: Methods - Data analysis, p. 11-12). The additional analyses on the broad time window are currently not included in the manuscript, but if the reviewer thinks they will be of added value to the reader, we are of course willing to include them in the supplementary materials.

Regarding the apparent reduction in HbO_2 and HHb for the high-risk infants in channel 25, we agree with the reviewer that this reduction may be interesting and potentially meaningful (see also our response to minor comment 7 by reviewer 2, page 18). From the time courses depicted in Figure 4, it seems that while the HbO_2 response for the low-risk infants steadily increases for the social condition, there is an initial decrease in the high-risk infants followed by an increase for both the social and non-social condition. To assess this initial response more closely, we inspected the data of channel 25 during a 3-7s time window post-stimulus

onset. In this window, we saw a significant increase from baseline in HbO_2 for the low-risk infants in the social condition ($t(11)=3.84, p=0.003$, uncorrected) but no other significant changes. For the high-risk infants, on the other hand, a significant reduction from baseline in HbO_2 was evident for the social condition ($t(15)=-2.18, p=0.046$, uncorrected) as well as for the non-social condition ($t(15)=-3.57, p=0.003$, uncorrected) but there were no differences from baseline for HHb for either condition (social: $t(15)=-1.37, p=0.191$/ non-social: $t(15)=1.91, p=0.075$). Since a significant reduction is only visible for one of the chromophores and HbO_2 and HHb are not decreasing in unison or mimicking each other, we would not consider this response as being an artifact. Rather, we suggest that it might be a meaningful response in the high-risk infants. However, as we would typically expect the HbO_2 response to increase during the presentation of the complex dynamic stimuli, it remains unclear what is driving this response in the high-risk infants and this phenomenon has to be investigated further in future studies using larger samples. Crucially, a similar initial decrease in the HbO_2 response has been observed by our colleagues specifically in a group of at-risk infants that continue to develop ASD (Lloyd-Fox et al., under review) suggesting that this initial response may be meaningful. We extended the discussion section of our paper to further elaborate on the time courses and discuss the potential meaning of this initial decrease in the HbO_2 response in the high-risk infants (see Discussion 4th paragraph p.17-18).

1.4 Could the individual differences of infants' attention to the stimuli (e.g., continuous vs. on/off attention within a trial) have an effect on the data?

In order to investigate whether subtle differences in infants' attention may have influenced our results, we performed a more detailed analysis of the infants' attention to the stimuli (see also major comment 1 by Reviewer 2, page 9). We extracted the average Looking Time for each child for the social, non-social, and baseline blocks and investigated group and condition differences as well as interaction effects between group and condition (see supplementary analyses 1). Importantly, we did not find any effect of group suggesting that visual attention was similar between the high- and low-risk infants. Although other individual differences in visual attention may theoretically be possible, we consider this very unlikely. In most cases, the infant either looked at the stimulus display for (almost) the entire trial continuously or did not look at the display at all. We did not encounter a lot of switching of attention, but rather saw that infant's lost interest completely. Given that the overall number of trials as well as the Looking Time did not differ between the low- and high-risk infants, we are confident that group differences in the infants' attention did not influence the current results.

2. The Data Analysis section stated that both HbO2 and HHb concentration changes were investigated, but the results do not mention any HHb results.

We indeed analyzed both HbO_2 and HHb concentration changes, but there were no significant HHb changes for any of the analyzed channels for neither condition or group. This is now stated explicitly in the results section of the manuscript (see: Results, p. 13, 14).

3. If channels 22 and 25 are positioned in spatial proximity and interpreted to reflect activity of the same pSTS-TPJ region, what would be the reason for their disparate results within and between the two subject groups?

Figure 4 suggests that changes in the HbO2 response to social stimuli could have been present at both channels, and the lack of statistical significance could be attributed to low power due to a small sample size. Similarly, while the results for sibs-ASD report an "increase in HbO2 concentration for the nonsocial dynamic condition in channel 22", it is not clearly visible in Figure 4. It would be informative to report the effect sizes for the observed condition and group differences, and also to test whether there was a significant difference in signal between channels 25 and 22.

Differences between channel 22 and channel 25

We agree with the reviewer that we would not necessarily expect disparate results for the two channels within and between the two groups. It is indeed likely that the relatively small sample sizes - in particular for the control group - reduced our power to detect differences. However, next to the sample size, our strict analysis criteria have also influenced our findings, resulting in a reduced number of channels that were considered significant.

For these reasons, we agree with the reviewer that the reported activation pattern for channel 25 and 22 may not be substantially different. In order to investigate this further, we followed the reviewer's suggestion and assessed whether there were differences in the activation of the two channels. As stated in the

supplementary analysis 2, “we performed a 2x2 repeated measures ANOVA for each of the infant groups separately, using Channel (Ch22, Ch25) and Condition (social, non-social) as within subject factors. For the low-risk group, we found a main effect of Condition ($F(1,10)=10.46$, $p<0.01$). As expected, responses elicited by the social condition were overall larger than responses to the non-social stimuli. The main effect of channel ($F(1,10)=2.31$, $p=0.16$) and the interaction effect were not significant ($F(1,10)=0.50$, $p=0.50$) for the low-risk infants. For the high-risk group, there was no significant main effect of condition ($F(1,14)=1.27$, $p=0.28$) or channel ($F(1,14)=2.56$, $p=0.13$), and the interaction effect did not reach significance either ($F(1,14)=2.53$, $p=0.13$). These results suggest that for both infant groups, the two channels did not differ significantly from each other, indeed hinting at no substantial difference in the responses of the two channels” (see supplementary analysis 2).

We have extended parts of the discussion to consider limitations with respect to power and our sample size in more depth and we have included a section that discusses the channel differences (see: Discussion, 5th paragraph, p. 18). We also added the results of the above-mentioned channel analysis in the supplementary material of the manuscript (see supplementary analyses 2).

Effect sizes of the group and condition differences

We would like to stress the fact that we did find significant group differences despite the relatively small sample and the strict analysis criteria. To further aid the interpretation of these results, we followed the reviewer’s suggestion and calculated the effect sizes for the condition and group effects. For both of our main results (i.e. the group difference for the social condition in channel 25 and the condition difference for the low-risk group in channel 25), we found a Cohen's *d* larger than 0.8 which suggests that both effects were large. The information on the effect sizes has been added to the results section of the manuscript (see Results, page 13-14).

4. The discussion section overstates the results. While the sibs-ASD group did not show a statistically significant change in HbO2 for the social stimuli, Figure 4 argues against them not showing “any social-specific activation”.
It is also surprising that the authors did not examine any associations between the fNIRS data and the behavioral measures of social-communicative functioning, as such analyses could help with the functional interpretation of the results. Given that the study sample comes from a larger longitudinal study, some relevant behavioral assessments are likely available.
In the absence of any brain-behavior association, the conclusion about “an early social processing deficit” in sibs-ASD is overly strong as not all brain differences translate into observable behavioral limitations, and most of the 16 sibs in this study may develop typically. Also, the discussion fails to consider the possibility of the observed fNIRS difference in the small sample at 5 months not being related to any later outcomes (e.g., Merin et al. (2007) found differences in gaze patterns in 6-month-old sibs-ASD, which later turned out not be predictive of ASD symptoms or severity as shown in Young et al. (2009)).
We followed the reviewer’s suggestions and adapted the discussion section to provide a more nuanced interpretation of the results. We agree that the word “deficit” may be misleading to the reader and we now describe our findings as processing “differences” (please see also major comment 2 by Reviewer 2, page 10). In addition, we expanded our discussion section to go into more depth on the possibility that our results may not be related to a later ASD diagnosis (see: Discussion, 6th paragraph, p. 19).

We further agree with the reviewer that it would be interesting to relate the neural activation patterns from the current study to behavioral markers of social development. However, such a link was beyond the scope of the current paper which aimed to replicate and extend previous neuroimaging findings. Behavioral data, such as parent-child interaction observations, was collected as part of our longitudinal study, but this data is still being processed and will become available only at a later point in time due to the laborious coding and analysis processes. It is also noteworthy that most behavioral studies fail to show differences in the first half year of development but rather report that consistent group differences only emerge later (Jones, Gliga, Bedford, Charman, & Johnson, 2014; Jones & Klin, 2013). The use of neuroimaging studies may enable us to detect deviant cortical activation prior and potentially independent of these behavioral symptoms.

Minor concerns:

1. *How the presence/absence of the ASD diagnosis in the siblings was determined?*

EJN European Journal of Neuroscience FENS Federation of European Neuroscience Societies

Families were included in the high-risk sample if at least one older sibling of the participant had received a clinical diagnosis within the autism spectrum. For all older siblings, a clinical report was available to the research team that was used to confirm the diagnosis of the child. Due to practical reasons, the older siblings were not diagnosed independently by our own research team. The manuscript has been adapted to clarify our procedures (see: Methods-Participants, p. 7).

2. *Was audio included with the visual stimuli?*

There was no audio included with the visual stimulus presentation. Our Social dynamic condition was the same as the visual-social condition by Lloyd-Fox et al. (2013) and the non-social dynamic stimuli design was similar to Lloyd-Fox et al. (2009), also containing no sound. This aspect of the stimulus design is now more clearly explained in the methods section of the manuscript (see: Methods-Stimulus Material, p. 8).

3. *Could the authors clarify why they opted to use multiple t-tests vs. a mixed model ANOVA?*

The analysis method we used was based on the previous studies (Lloyd-Fox et al., 2009, Lloyd-Fox et al. 2013). The reviewer is right in suggesting that a mixed model ANOVA would have been a very suitable alternative way of analyzing the current data. However, as we aimed to replicate previous findings, we decided to stay with the original way of analyzing the data by means of using multiple t-tests. As we are well aware that the use of multiple t-tests increases the risk of false positives, we decided to correct for multiple testing using False Discovery Rate methods. This way, we were able to follow the original data analysis and presentation, while correcting for the downsides of multiple t-testing.

Response to Reviewer 2

Major concerns:

1. This paper did not sufficiently address the potential influence of differences in looking patterns or attention to stimuli between the HR and LR groups. The authors attempted to control for attention by ensuring that groups had comparable numbers of trials included/excluded based on a criteria of 60% attention. Please provide explanation and justification for this choice of criteria as well as how attention criteria were operationalized. Without this clarification, it is unclear whether there may have been differences in the distribution of attention throughout the trial. For example, depending on if the 60% attention threshold was met by looking at the beginning, the middle, the end, or randomly interspersed glances throughout the 8-10s trial, these looking pattern differences could account for group differences in brain activation. Please calculate percent of time looking and number of looks for both groups for each condition to identify group differences, condition differences, and interaction effects.

We employed a cut-off ensuring that trials were only included if the infant had watched the display for 60% of the time during the dynamic stimulus presentation as well as 30% of the time during the pre- and post-stimulus static baseline. These values were chosen based on the previous study that we aimed to replicate and extend (Lloyd-Fox et al., 2013). For each block, it was continuously coded whether the infant was watching the screen or not and consecutively it was determined whether the infant's attention for each block was within the set criteria. In most of the cases infants either looked at the stimulus display for (almost) the entire trial continuously or did not look at the display at all. We did not encounter a lot of switching but rather saw that infants lost interest completely resulting in the rejection of the corresponding trial (see also major comment 1.4 of Reviewer 1, page 4).

However, we understand that the number of trials may not give sufficient information about potential group differences in visual attention that may have influenced our results. Therefore, we followed the reviewer's suggestion and re-analyzed the video coding data. As stated in the supplementary analysis 1, "we determined the percentage of looking for all of the social, non-social, and baseline blocks and compared the average looking time between blocks and between the high- and low-risk infant groups". An overview of the average looking time per block can be found in the table below (T1). "Importantly, infants' average looking time for the social and non-social stimuli exceeded 80% which is comparable to previous studies (Shimada & Hiraki, 2006) and suggests that infants were generally very attentive in the current study."

To assess condition differences, group differences and interaction effects, we performed a 3x2 repeated measures ANOVA with Block (social vs. non-social vs. baseline) as within subject factor and Group (High-risk vs. Low-risk) as between subject factor.

EJN European Journal of Neuroscience FENS Federation of European Neuroscience Societies

There was no main effect of Group ($F(1,27)=0.31$, $p=0.58$) and no interaction between Group and Block ($F(2,54)=1.25$, $p=0.30$). We found a significant main effect of Block ($F(2,54)=27.08$, $p<0.01$). Paired-sample t-tests showed that there was no difference between Looking Time during social and non-social blocks ($t(28)=0.81$, $p=0.43$) but that infants' Looking Time was lower for the baseline blocks compared to the social ($t(28)=5.65$, $p<0.01$) and non-social ($t(28)=7.56$, $p<0.01$) blocks. Given the nature of the stimuli (dynamic videos vs. static baseline stimuli) a difference between looking time between the two dynamic conditions and the baseline is expected. More importantly, the absence of a difference between the two conditions and between the two groups suggest that attentional effects are unlikely to have influenced the current results" (see supplementary analysis 1). These additional analyses have been added to the manuscript in the form of supplementary material.

	Social	Non-social	Baseline
Low-risk	86.84 (10.34)	81.87 (11.51)	70.67 (11.62)
High-risk	81.12 (10.42)	82.19 (12.85)	69.79 (13.78)
Total	83.68 (10.60)	82.05 (12.05)	70.18 (12.64)

Table T1. Overview of the average Looking Time per Block and infant group. Infants' Looking Time was shorter for baseline blocks compared to both the social and the non-social blocks. There was, however, no difference in Looking Time between the social and non-social blocks and there were no group differences for any of the three

2. The conclusion that the findings provide compelling evidence for an early social processing deficit is not supported by the experimental findings. Rather, phrasing the findings as an early social processing difference or abnormality would be more appropriate, as deficit connotes an impairment that is not demonstrated. Given that the fNIRS headgear is restricted to frontal and temporal lobes of the brain, HR infants could be discriminating between social and non-social stimuli in brain regions that were not examined in the present study.

We followed with the reviewer's remark and we adapted the manuscript, rephrasing parts our discussion and conclusion accordingly. We agree that the word deficit may be overstating the results and misleading for the reader we now describe our findings as processing "differences" (please see also major comment 4 by Reviewer 1, page 6-7).

3. *In the analysis section, the expectation of activation peak during 10-14 second time window is based on an in press paper. Please confirm that this time window is appropriate for both high risk and low risk infants in this sample.*

Our analysis window was indeed based on Lloyd-Fox et al. (2016), as their study suggests that such a narrower window around the peak of the activation would result in more robust activation compared to a broader window of for instance 8-14s. Inspecting the development of the cortical responses shown in Figure 4, the narrow window we used indeed surrounds the peak of the responses in the low-risk infants for both channels and in the high-risk infants for channel 22. Although the high-risk infants do not show a pronounced peak in HbO_2 in channel 25, the analysis window still seems to include the maxima of the responses for this channel (for further discussion of channel differences see minor comment 7, page 17-18). Based on this, we do believe that our analysis window was appropriate for the current dataset.

Importantly, previous research (Lloyd-Fox et al., 2013; Lloyd-Fox et al., under review) suggests that while the onset and strength of the haemodynamic response may differ between low- and high-risk infants, the latency of the time to peak appears to align with the length of the stimulus period in both groups. Furthermore, we see this pattern to be the case in many other studies of low-risk infants (though we admit there is less data available for high-risk infants). Therefore, we do not believe that the low- and high-risk infants would require different analysis windows. The previous study by Lloyd-Fox and colleagues (2013) and the companion study of the current paper (Lloyd-Fox et al., under review) used similar analysis windows around the peak of the response for both groups and reported significant activation for low- and high-risk infants suggesting that cortical activation using those windows can be detected in both groups.

However, following this query and one from Reviewer 1, we conducted an additional analysis to exclude the possibility that our choice of time window may have hidden effects occuring earlier in time. Specifically, we performed an additional analysis using a very broad time window of 0-14s post-stimulus onset as window of interest (see also major comment 1.3 by Reviewer 1, page 3-4). Using this window, we still found a significant change from baseline for HbO_2 concentration changes to the social condition in the low-risk infants in channel 25 ($t(11)$=, $p<0.001$, FDR-corrected). In addition, we found a significant difference in this channel between social and non-social condition in the low-risk infants ($t(11)=-3.09$, $p=0.01$) and for the social condition between the low- and high-risk group ($t(26)=-3.07$, $p=0.01$). These results are in line with the findings reported in the main manuscript and suggest that the main conclusions of our paper are not merely a result of a narrow analysis window but remain unchanged, even if a very broad time window is used. Importantly, and in line with a recent study by Lloyd-Fox et al. (2016), a narrower time window around the peak as used in the main analysis resulted in more significant channels suggesting that it indeed increased the sensitivity to detect cortical activation.

We have extended our description of the choice of analysis window in the methods section (see: Methods - Data analysis, p. 11-12). The additional analyses on the broad time window are currently not included in the manuscript, but if the reviewer thinks they will be of added value to the reader, we are of course willing to include them in the supplementary materials.

4. *Stimuli set:*

4.1 *a) Control condition doesn't appear adequate for purpose of the paper. Can two baselines work (i.e. static social and static non-social) to control for both social and non-social conditions? Or the static baseline could be more neutral such as a crosshair?*

We acknowledge the reviewer's point that other control conditions may be of added value to further investigate the processing differences in future studies, however they do also come with their own drawbacks. For example, a social static control condition may be useful to disentangle whether it is especially complex social stimuli (like the dynamic stimuli used in the current study) that are processed differently or whether diminished responses in high-risk infants can also be observed for static stimuli, however the introduction of two baselines would have required the presentation of a greater number of trials which may have led to increased participant drop out. Furthermore, a static crosshair baseline, though desirable from a neutral perspective, would be unsuitable in a block design with this aged participants as they would become restless and not attend to the study for a sufficient number of trials (while also introducing additional motion artifact in the data).

Moreover, we do not believe that these additional conditions were strictly necessary for the current study and we are convinced that the control condition we applied was adequate for its purpose. The previous study by Lloyd-Fox et al. (2013) has shown that social dynamic stimuli elicit temporal cortex activation in typically-developing infants compared to infants at risk for ASD. Yet, since temporal cortex activity is known to be modulated both by motion as well as by social aspects of the stimuli, a motion control condition is required to disentangle the two possibilities. Our dynamic nonsocial condition is similar in movement properties, does not contain social aspects, and hence fulfills the requirements to control for motion-sensitive areas that might underlie the observed temporal cortex activity.

Thus, while we do agree that future studies could apply additional control conditions to further investigate the neural processing differences in more detail, we do believe that our control condition is sufficient to show that differences were indeed related to the social, rather than the dynamic, nature of the stimuli.

4.2 *b) & c) The amount of motion in the social and non-social stimuli is claimed to be matched. Please quantify the matching procedure. Was luminance matched?*

The matching of the stimulus videos was done based on visual inspection of the motion patterns of the distinct videos, but there was no further quantification of the matching criteria. Stimuli were recorded using the same black background and with the intention to be as similar as possible in terms of luminance and other low-level features while still being naturalistic and infant friendly. We did not explicitly test for differences in luminance. Although it is possible that small differences in luminance existed, we do not believe that our current results can be explained by these low-level differences. Firstly, the regions that show activation in the current study are limited to the posterior temporal cortex (pSTS-TPJ) which overlays areas that are activated primarily by motion and social aspects of the stimuli (Lloyd-Fox et al., 2009). Secondly, we have no reason to believe that small, potential, differences between the two conditions would affect low- and high-risk infants differentially and hence cause the observed group differences.

Furthermore, we did ensure that stimulus materials for the social and non-social condition were equally engaging for the infants. This was confirmed by the absence of condition differences in infants' attention (see supplementary analysis 1).

4.3 *d) Overall, the description of the stimuli set was not clear. It's not clear whether each dynamic block is a single video or a combination of multiple counterbalanced videos. Figure 1 could be labeled with "baseline," "dynamic social," and "dynamic non-social" labels to clarify the presentation of stimuli. The number of different static and dynamic stimuli needs to be clarified.*

We agree with the reviewer's suggestion and have adapted Figure 1 as well as the stimulus description to clarify the stimulus set and design. We added additional information on the nature of the stimuli and their presentation (see: Methods-Stimulus Material, p.8).

5. *Sample differences*

5.1 *a) Please examine potential differences between high risk and low risk groups in terms of sex distribution and discuss how this could influence interpretation of the results.*

As the reviewer suggested, we analyzed sex differences using a Chi-Square analysis. The analysis confirmed that there were no differences in the distribution of sex between the low- and high-risk infants ($\chi^2(1, N=29)=1.88$, $p=0.17$). As there was no group difference in the sex distribution, we do not believe that sex has influenced our current results or the interpretation thereof. A note on the absence of a difference in the distribution of sex has been added to the manuscript in Table 1 (p.7).

5.2 b) Please justify use of ELC as matching criteria. The inclusion of gross and fine motor seem less relevant; confirm that groups were specifically comparable on RL, EL, and VR.

We chose to test for differences in the ELC to assure that our groups were matched on their overall development. This is what has typically been done in previous research studying infants at-risk (see e.g., Lloyd-Fox et al., 2013; Luyster, Wagner, Vogel-Farley, Tager-Flusberg, & Nelson, 2011; Seery, Vogel-Farley, Tager-Flusberg, & Nelson, 2013). To ensure that there were no differences specifically related to the infants' social development, we followed the reviewer's comment and analyzed the MSEL data in more depth.

To test for group differences, we compared the groups for each of the sub-scales separately using independent sample t-tests. Importantly, we found no significant differences between the low- and high-risk infants for any of the sub-scales (GM: $t(27)=0.95$, $p=0.35$; VR: $t(27)=0.96$, $p=0.35$, FM: $t(27)=1.39$, $p=0.18$, RL: $t(27)=1.28$, $p=0.21$, EL: $t(27)=-0.55$, $p=0.58$). Based on this finding, it remains highly unlikely that our results can be explained by differences in the receptive or expressive language or visual reception. We included the results of the individual t-tests in the methods section of the revised manuscript (see Methods-Participants, 3rd paragraph, p. 8).

During these analyses, we discovered that the MSEL ELC score for one of the control participants had not been registered properly. This mistake has been corrected in the current manuscript. We would like to stress that this did not change any of the presented results or interpretation of the findings in any way.

5.3 c) Additionally, the nearly significant (p=.09) group difference on ELC raises a concern about cognitive differences accounting for the results for a sample of this size. Please confirm experimental effects exist independent of IQ differences.

Please note that due to the mistake we found in the MSEL data of one participant (see previous comment), the results of the t-test changed from being marginally significant to being non-significant ($t(27)=1.44$, $p=0.16$). However, we did follow the reviewer's suggestion and verified that our main effect was not modulated by differences in the infants' development. The main group differences that we found in the current study was the difference in the HBO_2 response to the Social stimuli in channel 25. In order to verify that differences in IQ did not affect this result, we conducted a Univariate Analysis of Covariance comparing group differences in HBO_2 concentration changes and entering the ELC score as a covariate. In line with our main analyses, we found a main effect of group at significance level ($F(1,26)=4.21$, $p=0.05$). We therefore do not believe that differences in overall development can explain our results. This additional analysis is currently not included in the manuscript. However, we are willing to include it if the reviewer thinks it will be of added value to the reader.

Minor concerns:

1. *In Table 1, please add ranges to age and ELC distributions.*

The range of the age and ELC distribution has been added to the manuscript (see: Table1 in Methods-Participants, p. 7).

2. *The introduction could be tightened up by avoiding generalities like "many researchers have studied" or "many studies have reported". Most of the introduction and discussion sections are based off of previous research from the authors and do not deeply engage with what has previously been done in the literature.*

We agree with the reviewer's suggestion and have edited the manuscript to avoid these generalities (see: Introduction, 1st, 2nd and 3rd paragraph, page 3-4).

3. *Please discuss the use of both HbO2 and HHB in analysis for a general audience.*

We have extended our description in the methods section to include a more detailed justification of analyzing both HbO_2 and HHb (see: Methods-Data analysis, p. 12).

4. *Clarify how sound was controlled for. If sound was used to regain the infants' attention these trials should have been excluded. Based on the author's previous paper, HR and LR groups may be responding differently to vocal and non-vocal stimuli, which could compromise the findings in the current paper.*

The dynamic videos itself did not contain any sound and attention getting sounds were always non-vocal (such as bells, or ringing). We did not exclude trials during which attention getters were played, but we do not believe this to be problematic for our results or interpretation for the reasons outlined hereafter.
Importantly, attention getting sounds were only played on few occasions and only if the infant disengaged from the screen and did not recover attention by him/herself. As mentioned above (see major concern 1, page 9), the infants' overall attention to our stimulus display was very good (reaching more than 80% on average for both conditions) and the use of attention getting sounds was therefore very limited. In addition, most infants either watched the stimulus presentation continuously (where no attention getter was played) or disengaged completely (resulting in the rejection of the trial).
On a more fundamental note, sounds were played only for a short duration and not presented continuously for the entire block as in the experimental manipulation from previous research cited by the reviewer. Furthermore, in line with previous studies (Lloyd-Fox et al., 2009, Lloyd-Fox et al., 2013), if a sound was used in a trial (with say social content) we ensured that we played a sound again in the next non-social trial to ensure that sounds were played equally often for all condition types prohibiting a bias in the amount of sounds played between the different blocks. Although trials during which attention getting sounds were played were not excluded from the analysis (following the procedures by Lloyd-Fox et al., 2009, Lloyd-Fox et al., 2013), due to their infrequent use, and these counterbalancing measures, we do not believe that this has influenced our results.

5. *Elaborate on the implications of the finding that HR infants have greater activation to non-social dynamic stimuli in relation to baseline.*

We have expanded the discussion section to further interpret these findings (see: Discussion, 1st paragraph, p. 15-16). We do not consider it surprising that we see a significant increase in HbO_2 from baseline for the non-social stimuli, as such an activation was also observed in the previous study by Lloyd-Fox and colleagues (2009) in typically-developing infants. We argue that this activation is likely due to the more engaging nature of the dynamic stimuli compared to the static baseline. "Although it is interesting that the activation to the non-social condition in the high-risk infants did survive FDR-correction whereas the social activation did not, it is important to note that we did not find significant differences between the social and non-social stimuli. Rather the time courses of the HbO_2 responses shown in Figure 4 appeared very similar for both conditions. These results suggest that that both conditions were processed similarly by the high-risk infants and that the socially-selective processing visible in the low-risk infants was diminished in the at-risk group" (see Discussion, 1st paragraph, p.16)

6. *Please justify the decision to further analyze channels 22 and 25 due to significantly greater activation during dynamic compared to static stimuli. Significantly greater activation in response to dynamic compared to static stimuli would suggest that the activation is in response to*

motion. This presents the confound with motion that the authors were intending to avoid.

We indeed chose to further analyze channels which showed an increase in HbO_2 and/or a decrease in HHb compared to baseline during either type of the dynamic stimuli. As the previous study by Lloyd-Fox and colleagues (2013) found that high-risk infants showed a stronger response to the non-social (auditory) stimuli compared to the social stimuli, we did not want to bias our analysis towards channels responding more strongly to the social stimuli. While the reviewer is correct that a mere response to the non-social condition may indeed likely be a response to motion, a (hypothetical) conditional difference for the high-risk infants showing a stronger response to the non-social condition compared to the social condition is of great interest in the current study. Such a conditional difference would not purely be due to motion (as this was matched between conditions) but could rather reflect a tuning towards the non-social stimuli in the high-risk infants. Importantly, such a difference could be present without the social condition being significantly different from baseline and would therefore not be detected if only channels responding to the social condition were selected for further analysis. Hence we decided to include both channels that showed activation for the social and non-social condition.

7. *In Figure 4, in channel 25 for high risk infants the HbO2/HHb isn't looking as expected. In previous work (Lloyd-Fox et al., 2013), the authors said "if HbO2 and HHb were either to increase or decrease significantly in unison, the signal was considered inconsistent with a haemodynamic response to functional activation, and not reported in analyses", but this looks like exactly what's happening in channel 25 for HR infants. This seems to call into question the findings of between-group differences, since those were only observed in channel 25.*

We appreciate the comment of the reviewer and agree that an additional clarification of our analysis procedures may be necessary. We indeed followed the same analysis criteria as Lloyd-Fox and colleagues (2013) and we would therefore also have rejected channels that showed this type of coupled (de)activation.
However, the decision to consider a channel as (in)valid is based on the results from the statistical analysis where the observed increase or decrease is evaluated against a statistical threshold rather than being based on the inspection of the channel's time course. In our main analysis, HbO_2 and HHb did not show a significant response in channel 25 for the high-risk group. This suggests that there were no significant HbO_2 or HHb changes from baseline during the analyzed time window and therefore there was no reason to mark this channel as containing an artefact and reject it from our analysis.
However, we do agree with the reviewer that the early response in the high-risk infants does not look as expected and therefore we performed an additional analysis on channel 25, assessing HbO_2 and HHb responses during an early 3-7s time window (see also our response to Reviewer 1 Major Comment 1.3, page 3). Using this time window, we found a significant increase from baseline in HbO_2 for the low-risk infants in the social condition ($t(11)=3.84, p=0.003$, uncorrected) but no other significant changes. For the high-risk infants, on the other hand, a significant reduction from baseline in HbO_2 was evident for the social condition ($t(15)=-2.18, p=0.046$, uncorrected) as well as for the non-social condition ($t(15)=-3.57, p=0.003$, uncorrected), whereas there was no difference from baseline for HHb for either condition (social: $t(15)=-1.37, p=0.191$/ non-social $t(15)=1.91$, $p=0.075$). As HbO_2 and HHb responses are thus also not mimicking each other in this early time window and are not both decreasing in unison we would not consider this channel as containing an artifact during this early time window.
However, it is striking that the high-risk infants show an initial decrease in HbO_2 visible for both dynamic conditions as we would expect HbO_2 to increase in response to the social and non-social stimulus blocks. It is currently unclear what this initial decrease might mean. As mentioned above, we do not consider this response to be an artifact but would rather argue that it may be a meaningful deactivation characterizing the high-risk infants. Importantly, our colleagues (Lloyd-Fox et al., under review) found a similar initial decrease in a group of high-risk infants that continued to develop ASD suggesting that this phenomenon may indeed be meaningful. However, we currently do not know its cause and further research using larger samples is needed to assess this further and investigate its significance. We have extended our discussion section to interpret the time courses in more depth and discuss the apparent initial deactivation visible in the high-risk infants (see Discussion 4th paragraph p.17-18).

References

Grossmann, T., Lloyd-Fox, S., & Johnson, M. H. (2013). Brain responses reveal young infants' sensitivity to when a social partner follows their gaze. *Developmental Cognitive Neuroscience*, *6*, 155–161. http://doi.org/10.1016/j.dcn.2013.09.004

Jones, E. J. H., Gliga, T., Bedford, R., Charman, T., & Johnson, M. H. (2014). Developmental pathways to autism: A review of prospective studies of infants at risk. *Neuroscience and Biobehavioral Reviews*, *39*, 1–33. http://doi.org/10.1016/j.neubiorev.2013.12.001

Jones, W., & Klin, A. (2013). Attention to eyes is present but in decline in 2-6-month-old infants later diagnosed with autism. *Nature*, *504*(7480), 427–31. http://doi.org/10.1038/nature12715

Lloyd-Fox, S., Blasi, A.,Pasco, G., Gliga, T., Jones, E., Murphy, D., Elwell, & C.E., Charman, T., & Johnson, M. H. and the B. T. (under review). Cortical signature of autism evident before six months of life.

Lloyd-Fox, S., Begus, K., Halliday, D., Pirazzoli, L., Blasi, A., Papademetriou, M., … Elwell, C. E. (2016). Cortical specialisation to social stimuli from the first days to the second year of life: A rural Gambian cohort. *Developmental Cognitive Neuroscience*. http://doi.org/10.1016/j.dcn.2016.11.005

Lloyd-Fox, S., Blasi, a, Elwell, C. E., Charman, T., Murphy, D., & Johnson, M. H. (2013). 6.Reduced neural sensitivity to social stimuli in infants at risk for autism. *Proceedings. Biological Sciences / The Royal Society*, *280*(1758), 20123026. http://doi.org/10.1098/rspb.2012.3026

Lloyd-Fox, S., Blasi, a, Mercure, E., Elwell, C., & Johnson, M. (2011). The emergence of cerebral specialization for the human voice over the first months of life., (August 2013), 37–41. http://doi.org/10.1080/17470919.2011.614696

Lloyd-Fox, S., Blasi, a, Volein, a, Everdell, N., Elwell, C., & Johnson, M. (2009). Social Perception in Infancy: A Near Infrared Spectroscopy Study, *80*(4), 986–999. Retrieved from http://discovery.ucl.ac.uk/141037/

Luyster, R. J., Wagner, J. B., Vogel-Farley, V., Tager-Flusberg, H., & Nelson III, C. a. (2011). Neural Correlates of Familiar and Unfamiliar Face Processing in Infants at Risk for Autism Spectrum Disorders. *Brain Topogr*, *24*(3–4), 220–228. http://doi.org/10.1007/s10548-011-0176-z.Neural

Seery, A. M., Vogel-Farley, V., Tager-Flusberg, H., & Nelson, C. a. (2013). Atypical lateralization of ERP response to native and non-native speech in infants at risk for autism spectrum disorder. *Developmental Cognitive Neuroscience*, *5*, 10–24. http://doi.org/10.1016/j.dcn.2012.11.007

Shimada, S., & Hiraki, K. (2006). *Infant's brain responses to live and televised action*. *NeuroImage* (Vol. 32). http://doi.org/10.1016/j.neuroimage.2006.03.044

2nd Editorial Decision 30 May 2017

Dear Ms. Braukmann,

Your resubmitted manuscript was reviewed by the two original external reviewers as well as by the Section Editor, Dr. Sophie Molholm, and ourselves.

The reviews collectively indicate that your experiments generated new and important information. However, there are a few issues that need to be clarified/resolved before we can go forward with your paper. Reviewer 2 asks for some additional information, which should be relatively trivial for you to add. Other than this, both reviewers are satisfied with your revision of the manuscript.

Please also attend to the following issues in your final version:
1. A graphical abstract is needed
2. Your referencing is still not in EJN style
3. The footnote still needs to be either removed or relocated to the main text.
4. Provide the full name of the ethics committee that approved the study procedures
5. Information on the data repository still needs to be moved to a separate data accessibility statement.
6. Author contributions need to be included in the manuscript itself
7. Fig 2: did the parents give permission for this image to be published? This needs to be explicitly stated.

If you are able to respond fully to the points raised, we would be pleased to receive a revision of your paper within 30 days.

Thank you for submitting your work to EJN.

Kind regards,

John Foxe & Paul Bolam
co-Editors in Chief, EJN

Reviews:

Reviewer: 1 (Sasha Key, Vanderbilt Kennedy Center, USA)

Comments to the Author
The authors addressed my previous concerns.

Reviewer: 2 (James McPartland, Yale Child Study Center, USA)

Comments to the Author
The authors have been thoroughly responsive to my critique. Most concerns are assuaged, and I consider the revised manuscript significantly improved. I have only a few additional recommendations:
In response to previous Major concern 4.3, regarding lack of clarity in the description of the stimulus set: The clarified figure was very helpful. Please expand the information in the text to provide information about the number of different stimuli for dynamic and static conditions. Please clarify whether participants were exposed to repeats of stimuli. It is clear that conditions were counterbalanced, but please clarify whether stimuli within conditions were counterbalanced or randomized in sequence of presentation.

The additional analyses have been added to the discussion; however, I recommend including them in the results and then reviewing them in discussion.

Regarding the matching of the stimuli (previous concern 4.2), please provide detailed information about how the amount of motion was matched (visual comparison of motion energy is insufficiently concrete). If, as described in the response to reviews, this was based on visual inspection alone, it is inaccurate to describe stimuli as matched. It would be more appropriate to describe what was done lest readers assume a more rigorous matching approach was applied.

Authors' Response 25 June 2017

Response to reviewer

1. In response to previous Major concern 4.3, regarding lack of clarity in the description of the stimulus set: The clarified figure was very helpful. Please expand the information in the text to provide information about the number of different stimuli for dynamic and static conditions. Please clarify whether participants were exposed to repeats of stimuli. It is clear that conditions were counterbalanced, but please clarify whether stimuli within conditions were counterbalanced or randomized in sequence of presentation.
We extended the description of the stimuli to include more details about the number of stimuli and their randomization. For the static baseline blocks, a random selection of 6 images out of a total of 19 stimuli was presented during each block. For the social and non-social dynamic blocks, there were 6 different stimulus videos in total and one of those videos was pseudo-randomly selected to be presented during each block (see Methods-Stimulus material, p.8)

2. *The additional analyses have been added to the discussion; however, I recommend including them in the results and then reviewing them in discussion.*

We followed the reviewer's recommendation and included the additional analyses in the results section. The complete description of the analysis assessing differences between channel 25 and 22 has been moved from the supplementary materials and is followed by the description of the additional analysis assessing the early decrease in HbO_2 for the high-risk infants. (see Results, p. 14-15; Discussion p. 18-19)

3. *Regarding the matching of the stimuli (previous concern 4.2), please provide detailed information about how the amount of motion was matched (visual comparison of motion energy is insufficiently concrete). If, as described in the response to reviews, this was based on visual inspection alone, it is inaccurate to describe stimuli as matched. It would be more appropriate to describe what was done lest readers assume a more rigorous matching approach was applied.*

The final selection of non-social videos was indeed based on the visual inspection of the motion energy of the different videos alone. Therefore, we followed the reviewer's suggestion and rephrased the corresponding paragraph in the methods section. In particular, we now describe our selection procedure in more detail and do not explicitly label the stimuli as matched (see Methods-Stimulus material, p.8, Discussion p.16)
