# Supplementary material for: Diminished socially selective neural processing in 5‐month‐old infants at high familial risk of autism
Source: Eur J Neurosci. 2017 Nov 22;47(6):720–8. doi: 10.1111/ejn.13751 (PMC5943701; doi:10.1111/ejn.13751)
Supplement: Supplementary file 2 — Table S1. Results from the one‐sample t‐tests assessing significant changes in HbO2 and HHb with respect to baseline in the low‐risk and high‐risk infant group. Appendix S1. Analysis of group and conditional differences in stimulus attention. [file EJN-47-720-s002.pdf]

Supplementary Material

A) Supplementary Table 1

Supplementary Table 1.

Results from the one-sample t-tests assessing significant changes in HbO_2 and HHb with respect to baseline in the low-risk and high-risk infant group. Channels reaching significance based on uncorrected p-values are shown for each of the contrasts together with their FDR-corrected values which were used to determine significance in the main manuscript. Ch represent the channel number and pFDR represents the corrected p-value using False Discovery Rate correction. FDR corrected values were calculated using the mafdr function implemented in Matlab and corrections were done for the number of channels (n=26) used within each contrast. Significant channels after correction are highlighted bold.

Low-risk infants

**Social Dynamic vs. Baseline**								**Non-Social Dynamic vs. Baseline**							
HbO2				HHB				HbO2				HHB			
Ch	*t*	*p*	*p_FDR*	Ch	*t*	*p*	*p_FDR*	Ch	*t*	*p*	*p_FDR*	Ch	*t*	*p*	*p_FDR*
9	2.37	0.039	0.122	12	-3.11	0.009	0.117	10	2.60	0.023	0.456	21	2.54	0.027	0.714
10	3.09	0.009	0.082	13	3.43	0.006	0.117	12	2.38	0.035	0.456				
12	3.48	0.005	0.059												
15	2.43	0.033	0.122												
14	2.53	0.028	0.122												
22	2.33	0.042	0.122												
23	2.62	0.024	0.122												
25	4.07	0.002	**0.048**												
26	2.31	0.039	0.122												

High-risk infants

**Social Dynamic vs. Baseline**								**Non-Social Dynamic vs. Baseline**							
HbO2				HHB				HbO2				HHB			
Ch	*t*	*p*	*p_FDR*	Ch	*t*	*p*	*p_FDR*	Ch	*t*	*p*	*p_FDR*	Ch	*t*	*p*	*p_FDR*
10	2.17	0.049	0.267	16	2.49	0.028	0.553	7	2.57	0.023	0.304	20	2.35	0.034	0.296
13	2.82	0.013	0.267					13	2.21	0.043	0.370	21	3.54	0.003	0.077
15	2.22	0.044	0.267					22	3.92	0.002	**0.040**	24	2.51	0.024	0.296
26	2.36	0.033	0.267												

B) Supplementary analysis: Analysis of Group and Conditional differences in Stimulus Attention

To ensure that our results were not confounded by group or condition differences in stimulus attention, we performed additional analyses on the video coding data. More specifically, we determined the percentage of looking for all of the social, non-social, and baseline blocks and compared the average looking time between blocks and between the high- and low-risk infant groups. An overview of the average looking time per Block can be found in the table below (Table BT1). Importantly, infants' average looking time for the social and non-social stimuli exceeded 80% which is comparable to previous studies (Shimada & Hiraki, 2006) and suggests that infants were generally very attentive in the current study.

To assess condition differences, group differences and interaction effects, we performed a 3x2 repeated measures ANOVA with Block (social vs. non-social vs. baseline) as within subject factor and Group (High-risk vs. Low-risk) as between subject factor. There was no main effect of Group ($F(1,27)=0.31$, $p=0.58$) and no interaction between Group and Block ($F(2,54)=1.25$, $p=0.30$). We found a significant main effect of Block ($F(2,54)=27.08$, $p<0.01$). Paired-sample t-tests showed that there was no difference between Looking Time during social and non-social blocks ($t(28)=0.81$, $p=0.43$) but that infants' Looking Time was lower for the baseline blocks compared to the social ($t(28)=5.65$, $p<0.01$) and non-social ($t(28)=7.56$, $p<0.01$) blocks. Given the nature of the stimuli (dynamic videos vs. static baseline stimuli) a difference between looking time between the two dynamic conditions and the baseline is expected. More importantly, the absence of a difference between the two conditions and between the two groups suggest that attentional effects are unlikely to have influenced the current results.

Table BT1. Overview of the average Looking Time per Block and infant group

	Social	NonSocial	Baseline
Low-risk	86.84 (10.34)	81.87 (11.51)	70.67 (11.62)
High-risk	81.12 (10.42)	82.19 (12.85)	69.79 (13.78)
Total	83.68 (10.60)	82.05 (12.05)	70.18 (12.64)
